# Sharing information across patient subgroups to draw conclusions from sparse treatment networks

**DOI:** 10.1002/bimj.202200316

**Published:** 2024-04-18

**Authors:** Theodoros Evrenoglou, Silvia Metelli, Johannes-Schneider Thomas, Spyridon Siafis, Rebecca M. Turner, Stefan Leucht, Anna Chaimani

**Affiliations:** 1 Center of Research in Epidemiology and Statistics (CRESS-U1153), Université Paris Cité, INSERM, Paris, France; 2 Department of Psychiatry and Psychotherapy, School of Medicine, Technical University of Munich, Munchen, Germany; 3 MRC Clinical Trials Unit, University College London, London, UK

**Keywords:** informative priors, mixed treatment comparisons, sharing information, sparse data

## Abstract

Network meta-analysis (NMA) usually provides estimates of the relative effects with the highest possible precision. However, sparse networks with few available studies and limited direct evidence can arise, threatening the robustness and reliability of NMA estimates. In these cases, the limited amount of available information can hamper the formal evaluation of the underlying NMA assumptions of transitivity and consistency. In addition, NMA estimates from sparse networks are expected to be imprecise and possibly biased as they rely on large-sample approximations that are invalid in the absence of sufficient data. We propose a Bayesian framework that allows sharing of information between two networks that pertain to different population subgroups. Specifically, we use the results from a subgroup with a lot of direct evidence (a dense network) to construct informative priors for the relative effects in the target subgroup (a sparse network). This is a two-stage approach where at the first stage, we extrapolate the results of the dense network to those expected from the sparse network. This takes place by using a modified hierarchical NMA model where we add a location parameter that shifts the distribution of the relative effects to make them applicable to the target population. At the second stage, these extrapolated results are used as prior information for the sparse network. We illustrate our approach through a motivating example of psychiatric patients. Our approach results in more precise and robust estimates of the relative effects and can adequately inform clinical practice in presence of sparse networks.

## 1 Introduction

Network meta-analysis (NMA) has become an essential tool for the comparative effectiveness research of healthcare interventions because it allows integrating all available information on a specific disease and usually provides estimates of relative effects with the highest possible precision (Chaimani et al., 2019; Nikolakopoulou et al., 2018). However, networks of interventions with limited data that fail to provide useful conclusions may arise under certain situations. A recent empirical study found 92 (7.4%) published NMAs that included more treatments than the number of studies in a sample of 1236 NMAs of randomized controlled trials (RCTs) with at least four interventions (Veroniki et al., 2021). These numbers suggest that the phenomenon of “sparse” networks, namely, networks with limited direct evidence in the form of few direct comparisons and/or few—1 or 2—studies per comparison, is not very rare in the literature. For example, this is often the case for “sensitive” subgroups of the population that cannot be easily included in trials, such as children, elder patients, or individuals with multimorbidity.

Results from such networks of interventions are accompanied with substantial uncertainty not only in the estimation of the relative treatment effects but also in the plausibility of the underlying assumptions because their formal evaluation is impossible (Dias et al., 2010). In addition, the large-sample approximations on which NMA models typically rely upon fail in the presence of only a handful of studies per comparison. Hence, lack of robustness and potentially limited reliability are common issues that might be encountered when analyzing sparse treatment networks (Yoon et al., 2021). To avoid these issues, it might seem reasonable to wait until more studies become available for the outcome(s) of interest. However, the pace of trial production in these contexts is usually very slow and the chance to obtain new study results shortly is low.

The use of external evidence in the form of informative prior distributions has been suggested previously in meta-analysis, either to achieve more accurate estimation of the heterogeneity parameter (Röver et al., 2019; Turner et al., 2019) or for the incorporation of non-randomized evidence (Efthimiou et al., 2017). Here, we introduce a new framework that allows sharing information between two networks of treatments that pertain to different subgroups of the population. Our framework refers to the case when only aggregate data are available; in the presence of individual participant data, other approaches may be used, such as population adjustment methods (Phillippo et al., 2020). Specifically, we use the results from a subgroup with a lot of direct evidence (i.e., many direct comparisons and many studies per comparison) forming a “dense” network to construct informative priors for the relative effects in the target subgroup forming a sparse network.

Information sharing across population subgroups is also common outside the context of NMA. For example, similar approaches are used in the so-called “basket trials,” where information is borrowed from large baskets to facilitate the estimation of treatment effects in smaller baskets (Parmar et al., 2017; Renfro & Sargent, 2017; Turner et al., 2022) involving subpopulations that are challenging to include in clinical trials. The idea of extrapolating information from a population with a lot of data to another with limited evidence has been discussed previously in an FDA report (U.S. Department of Health & Human Services Food & Drug Administration, 2016) that used adult data to draw conclusions for children for the case of individual studies. Extending these ideas to the meta-analytic setting would allow us to obtain more robust conclusions as individual trials are often underpowered (Salanti et al., 2008).

In this manuscript, we propose a two-stage approach where at the first stage, we synthesize the data from the dense network using a hierarchical NMA model. This includes a location parameter that shifts the distribution of the relative effects to make them applicable to the population of the sparse network. We also add a scale parameter that allows us to further downweight the external data and to reduce their influence. At the second stage, the results from the first stage are used as prior information in the analysis of the sparse network. We inform the location and scale parameters either through the data or using expert opinion. We illustrate our approach through an example examining the relative effects of antipsychotic treatments in two subgroups of patients: a target subgroup of children adolescents (CAs) that forms a rather sparse network and a subgroup of chronic adult patients with acute exacerbation of schizophrenia, referred here as “general patients” (GP), which forms a dense network.

## 2 Motivating Dataset

Our work is motivated by a recent article investigating the differences in the treatment effects among several subgroups of schizophrenia patients (Leucht et al., 2022). The aim of the article was to compare the subgroup of GP, defined as chronic patients with an acute exacerbation of positive symptoms, with more specific subgroups of patients: CA, first-episode patients, treatment-resistant patients, patients with negative symptoms, patients under substance abuse, and elder patients. In contrast to the dataset for GP, all these specific subgroups were informed by very limited direct evidence, and therefore, the original authors, who acknowledged the problems of analyzing sparse networks, only used pairwise meta-analysis. To illustrate our approach, we use the data from two of the above subgroups: CA and GP. The former is considered the target subgroup that forms a sparse network and the latter is the only available subgroup with abundant direct evidence (forming a dense network) that can be safely used to elicit informative priors for CA.

**Figure 1:**
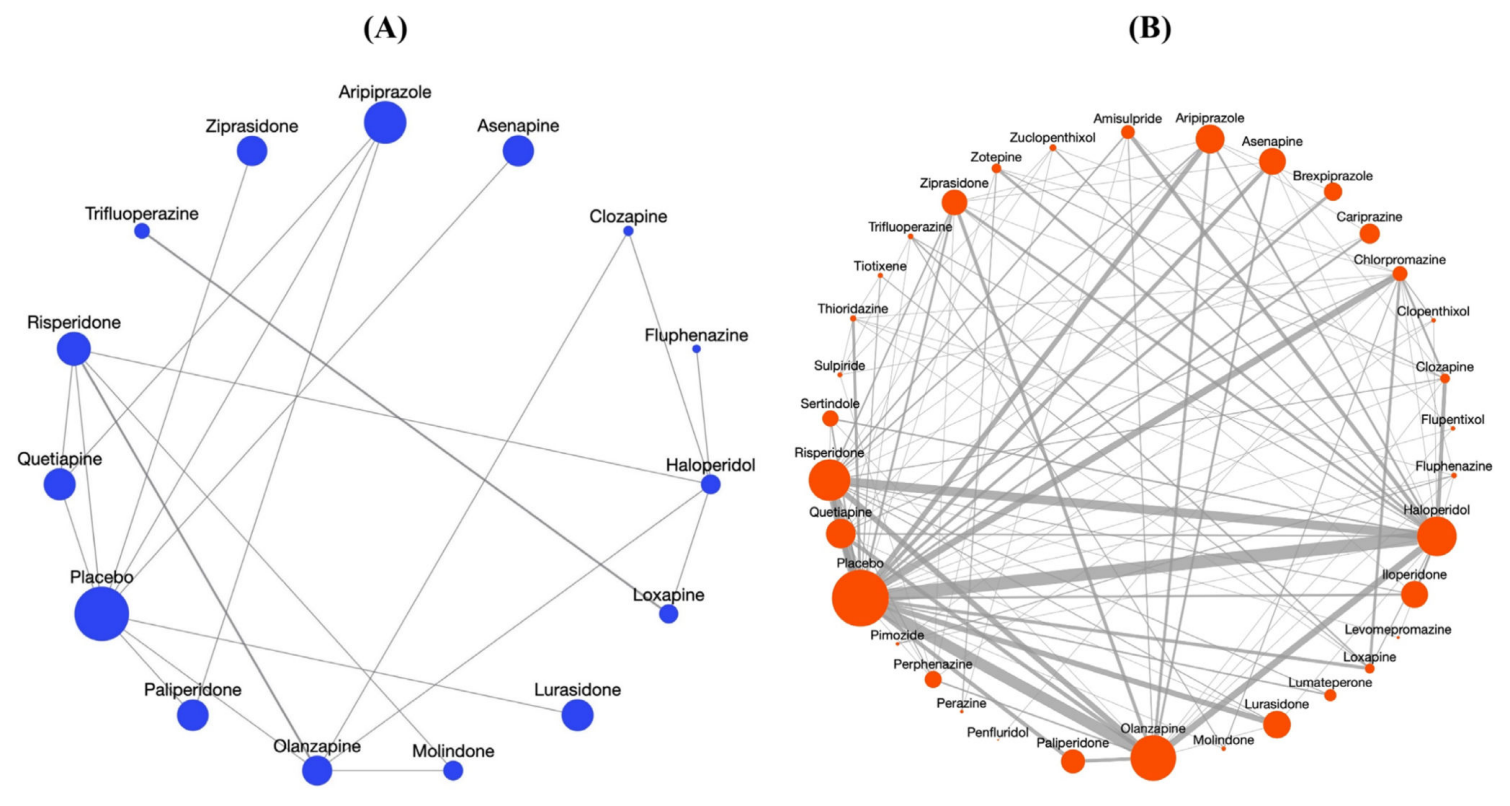
Sparse network for the population of CA (Panel A) and informative network for GP (Panel B). The size of the edges (lines in the networks) is proportional to the number of studies that compare the respective treatments, whereas the size of the nodes is proportional to number of patients who received each treatment.

The CA network comprises 19 RCTs published between 1973 and 2017 comparing 14 antipsychotics and placebo, and providing direct information for 21 out of 105 possible comparisons (Figure 1a). Out of these 21 comparisons, two include two RCTs and the rest include one RCT only. In addition, many of these RCTs are small, with a median study sample size of 113 patients. The pace of new RCT production for this subgroup is very slow (on average, one RCT every 2.3 years), which confirms the difficulty of conducting RCTs for this group of patients. On the other hand, the network for GPs contains 255 RCTs comparing 33 antipsychotics and placebo, and forming 116 direct comparisons (Figure 1b). We also used the community detection algorithm proposed by Law et al. (2019) to examine how sparse or dense the two networks are (Online Appendix 1). The conclusions from this approach are in agreement with what can be obtained by descriptive characteristics and visual inspection of the network diagrams. Specifically, almost all treatments except one (Molindone) in the GP network appear to be very close to each other and thus well identified (Appendix 1, Figure 1). In contrast, six treatments in the CA network appear isolated that reveals that at least 40% of this network is not well identified.

The two networks are distinct, which means that they do not have any RCT in common. However, some drugs are included in both networks; this is a necessary condition to allow sharing information between them. The outcome of interest is the reduction of the overall schizophrenia symptoms that is measured in a continuous scale. Following the original article, we used the standardized mean difference (SMD) as effect measure because the different RCTs have used different scales.

## 3 Methods

### 3.1 Notation and general setting

Suppose that we have a set of *N* = *n*_1_ + *n*_2_ studies. Studies {1, 2, …, *n*_1_} inform the target population subgroup *P*_1_ and form a sparse network, while the rest {*n*_1_ + 1, *n*_1_ + 2,…, *N*} inform a different population subgroup *P*_2_ and form a dense network. Suppose also that *T*_1_ treatments have been evaluated for population *P*_1_ and *T*_2_ treatments for population *P*_2_. We denote with *T*_*a*_ = *T*_1_ ∪ *T*_2_ the set of all available treatments and with *T*_*c*_ = *T*_1_ ∩ *T*_2_ the set of common treatments included in both networks; the latter is the group of treatments for which information can be extrapolated from *P*_2_ to the target subgroup *P*_1_. Each study *i* provides the observed mean (change score/endpoint) $y_{it_{k}}$ for the treatment *t_k_* ∈ *T*_*a*_ of arm *k* with *k* = 1, …, *K*_*i*_ and *K*_*i*_ the total number of arms in *i*. For simplicity, in what follows we write the $y_{it_{k}}$ as *y*_*ik*_, its variance $sd_{ik}^{2}$ , and the respective “true” mean is denoted as *θ*_*ik*_. We consider treatment *j* = 1 as the common network reference for both *P*_1_ and *P*_2_ and the relative effects *µ*_1*j*_ (*j* > 1, *j* ∈ *T*_*a*_) as the basic parameters. For every study, we arbitrarily choose the treatment of arm *k* = 1 (*t*_1_) as the study-specific baseline treatment.

### 3.2 Models for sharing information between two population subgroups

Different models stem from different assumptions about the relation between the two subgroups *P*_1_ and *P*_2_. We start with the description of the standard hierarchical NMA model that in the present setting synthesizes the two subgroups in a naïve way, namely, as if they were equivalent. Then, we move to more plausible NMA models in which we incorporate a location parameter acknowledging the difference between *P*_1_ and *P*_2_ and a scale parameter aiming to increase the uncertainty of the studies in subgroup *P*_2_.

#### 3.2.1 Naïve synthesis

In this approach, the two subgroups are combined together as if all participants were coming from the same population *P*. In other words, this approach makes the strong assumption that *P*_1_ and *P*_2_ are equivalent, namely, *P*_1_ ≡ *P*_2_ ≡ *P*. Hence, for every study *i* = 1, … *N* and treatment *t_k_* ∈ *T*_α_, the observed means follow a normal distribution

(1) $$y_{ik}∼\text{N}\left(\theta_{ik},sd_{ik}^{2}\right).$$

Then, the underlying SMD between every treatment $t_{2},t_{3},\ldots ,t_{K_{i}}$ versus the baseline treatment *t*_1_ is

(2) $$\delta_{i,1k}=\frac{\theta_{ik}-\theta_{i1}}{sd_{i}^{pooled\;}},(k>1),$$

with $sd_{i}^{pooled\;}=\sqrt{{\text{Σ}}_{k=1}^{K_{i}}\frac{n_{ik}sd_{ik}^{2}}{n_{ik}-K_{i}}}$ the between-arm standard deviation. Under the random-effects assumption, the underlying relative effects are assumed to follow a multivariate normal distribution

(3) $$\delta_{i}∼{\text{N}}_{K_{i}-1}(\mu ,\text{Σ}),$$

where $\mu =\left(\mu_{12},\ldots ,\mu_{1K_{i}}\right)$ is the vector of the summary relative effects and **Σ** the between-study variance–covariance matrix with entries τ^2^ (the common heterogeneity variance across comparisons) in the diagonal and $\frac{\tau^{2}}{2}$ in the off-diagonal. Finally, the transitivity assumption for every pair of treatments *j, l* ∈ *T*_*a*_ (*j, l* > 1) implies that *µ*_*jl*_ = *µ*_1*l*_ − *µ*_1*j*_.

Non-informative prior distributions are usually employed for every *µ*_1*j*_ and τ, such as N(0, 10000) and the half-normal HN(1), respectively.

Apart from the strong assumption about the similarity of the two subgroups, a further problem in the present setting is that the final results for the joint population *P* will be dominated by the dense network (*P*_2_) rather than from the target subgroup *P*_1_. The same would apply if the results of the dense network were used directly as prior information for *P*_1_. To avoid this issue, in the proposed approach, we extrapolate the results from *P*_2_ to *P*_1_, acknowledging differences between the two subgroups, before forming informative prior distributions for *P*_1_.

#### 3.2.2 Using the external subgroup *P*_2_ to construct informative priors for the target subgroup *P*_1_

This is a two-stage approach where at the first stage, we extrapolate the results from the dense network of subgroup *P*_2_ to *P*_1_, and at the second stage, we use predictions from this extrapolation to form prior distributions for the analysis of *P*_1_.

##### First stage: Extrapolating external results to the target population subgroup

In contrast to the naïve synthesis, the main assumption here is that the two subgroups have some underlying differences in the treatment effects that can be considered as a different location of their outcome distributions. To incorporate some uncertainty about this assumption, in Equation (1), we introduce a scale parameter *w*_*i*_ ∈ (0, 1] that inflates the variance of each *y*_*ik*_ in subgroup *P*_2_ for each *i* = *n*_1_ + 1, *n*_1_ + 2,…, *N* (Baker & Jackson, 2013). This modifies Equation (1) into

(4) $$y_{ik}∼\text{N}\left(\theta_{ik},\frac{sd_{ik}^{2}}{w_{i}}\right).$$

Equation (4) implies that the parameters *w*_*i*_ only affect the variance of the the mean scores *y*_*ik*_ and not the true study-specific SMDs that are still the same as in Equation (2). Assuming that $\beta_{1j}=\mu_{1j}^{P_{2}}-\mu_{1j}^{P_{1}}$ for each {1, *j*}, *j* ∈ *T_c_*, the location parameters ***β*** that aim to shift the original distribution of the SMDs in *P*_2_ toward the distribution of *P*_1_ are then added in Equation (3):

(5) $$\delta_{i}∼{\text{N}}_{{\text{K}}_{\text{i}}-1}\left(\mu^{P_{2}}-\beta ,\text{Σ}\right),$$

with $\beta =\left(\beta_{12},\ldots ,\beta_{1K_{i}}\right)$ being the vector of the “true” differences between the comparison-specific SMDs of the two sub-groups. The extrapolated means $\mu^{*}=\mu^{P_{2}}-\beta$ for population *P*_2_ in Equation (5) are then considered similar to those expected for population *P*_1_. Note that Equation (5) could be equivalently written as $\delta_{i}∼{\text{N}}_{{\text{K}}_{\text{i}}-1}\left(\mu^{P_{2}}+\beta ,\Sigma \right)$, if we had assumed that $\beta_{1j}=\mu_{1j}^{P_{1}}-\mu_{1j}^{P_{2}}$.

By fitting this modified NMA model, we obtain the extrapolated SMD estimates ${\hat{\mu }}_{jl}^{*}$ for every pair of treatments *j, l* ∈ *T*_*c*_. Subsequently, the predictive distributions (Higgins et al., 2009) of $\mu_{jl}^{new\;}$, namely, $\text{N}\left(\mu_{jl}^{*},{\left(\tau^{P_{2}}\right)}^{2}\right)$ are used at the second stage as prior distributions for *P*_1_. Note that the parameter ${\left(\tau^{P_{2}}\right)}^{2}$ represents the heterogeneity across the studies in *P*_2_.

##### Second stage: NMA of the target subgroup using informative prior distributions for relative effects

Here, the model of Section 3.2.1 is used for *i* = 1, …, *n*_1_. The key difference is that for the *µ*_1*l*_ s, we use as prior distribution $\mu_{1l}^{P_{1}}∼\text{N}\left({\hat{\mu }}_{1l}^{new\;},\mathrm{var}\left({\hat{\mu }}_{1l}^{new\;}\right)\right)$ where ${\hat{\mu }}_{1l}^{new\;}$ and $\text{var}\left({\hat{\mu }}_{1l}^{new\;}\right)$ are the posterior mean and variance of $\mu_{1l}^{new\;}$ , respectively. The use of informative priors is expected to improve the precision in the final NMA estimates without dominating the analysis. This is because the priors are constructed at the first stage by extrapolating the results of the dense network to those expected for the target sparse network.

#### 3.2.3 Informing the location and the scale parameters

##### Constructing prior distributions for *β*s using the data

To obtain an estimate of the difference in the outcome distributions between the two subgroups, we first use the estimates $u_{1j}^{P_{1}}$ and $u_{1j}^{P_{2}}$ obtained from separate pairwise meta-analyses for each population and each pair of treatments {1, *j*}, *j* ∈ *T_c_*. To allow the estimation of heterogeneity for every comparison in *P*_1_, here we assume a common comparison-specific heterogeneity across the two subgroups ${\left(\sigma_{1j}^{P_{1}}\right)}^{2}={\left(\sigma_{1j}^{P_{2}}\right)}^{2}=\sigma_{1j}^{2}$. Using the difference of these estimates $d_{1j}=u_{1j}^{P_{2}}-u_{1j}^{P_{1}}$, we construct prior distributions for the parameters *β*_1*j*_, namely,

(6) $$\beta_{1j}∼\text{N}\left(d_{1j},\text{var}\left(d_{1j}\right)\right).$$

For treatment comparisons not evaluated in *P*_2_, we use a non-informative normal prior N(0, 10000).

##### Constructing prior distributions for *β*s using expert opinion

Elicitation of expert opinion can be undertaken with several methods such as face-to-face interviews, software tools, or questionnaires. The pooled change score of each treatment *j* ∈ *T*_*c*_ in *P*_2_ can be used as a reference for the experts who need to provide an “estimate” for the change score in *P*_1_. Suppose that *x*_*hj*_ is the change score and *sd*_*hj*_ the standard deviation provided by the expert *h* = 1, 2, …, *H* for treatment *j* ∈ *T*_*c*_. Let also *γ*_*h*_ denote the overall confidence of each expert to provided opinions. To combine the estimates provided by different experts, we can then use the following meta-analysis model:

(7) $$x_{hj}∼\text{N}\left(c_{hj},\frac{sd_{hj}^{2}}{\gamma_{h}}\right),$$

(8) $$d_{hj}∼\text{N}\left(\xi_{j}^{P_{1}},\sigma^{2}\right),$$

with $d_{hj}=\frac{c_{hj}}{\text{med}\left(sd_{i}^{poled\;}\right)}$, ∀*i, h, j* the standardized values of *c*_*hj*_. Given that the pooled standard deviation cannot be elicited by experts, the median across the studies in the data is used. Note that standardization is not necessary when all studies in the data use the same scale. Then, the pooled SMDs can be obtained as $u_{1j}^{P_{1}}=\xi_{j}^{P_{1}}-\xi_{1}^{P_{1}}$ and the priors for the parameter *β*_1*j*_ is constructed as in Equation (6).

##### Prior distributions for the scale parameter

Dividing the variances in Equation (4) by *w*_*i*_ can be seen as a special case of the power prior method where a specific power is chosen for the likelihood of each study (Chen & Ibrahim, 2000; Efthimiou et al., 2017; Ibrahim et al., 2015). Values of *w*_*i*_ close to 0 reflect a serious downweight of the evidence coming from the external subgroup *P*_2_, whereas values close to 1 imply a mild downweight. Typical choices of prior distributions for the scale parameter can be the beta or the uniform distribution. The parameters of these distributions should be chosen to reflect the prior beliefs regarding the specific characteristic according to which the downweight takes place. For example, a Beta(3, 3) can be used for cases where a moderate downweight needs to apply for the external information. This distribution places its mass around 0.5 with 95% range equal to [0.15, 0.84] thus reflecting more uncertainty about the magnitude of our downweighting. Left-skewed beta priors (e.g., Beta(1, 6)) can be used for weight values close to 0, while on the other hand, right-skewed beta priors (e.g., Beta(6, 1)) can be used for weight values close to 1. The previous downweighting schemes can be achieved with other types of prior distributions. For example, a Unif (0.4, 0.6), a Unif (0.1, 0.3) or a Unif (0.8, 1) can be used for moderate, serious, or mild downweight, respectively. Finally, it is generally not recommended to use priors for the scale parameter that can allow for values larger than 1. This could further increase the impact of external data and enable their dominance in the final NMA estimates.

## 4 Application

### 4.1 Implementation

We consider throughout CA being the target population subgroup *P*_1_ and GP the external subgroup *P*_2_. We applied in total nine models six of which refer to the combinations of the different prior distributions for the location (*β*_1*j*_) and scale parameters (*w*_*i*_).

1. Naïve synthesis model for CA and GP with non-informative priors.
2. NMA for CA with informative priors from GP.
  - data-based prior distributions for the location parameters with
    (i) no downweight for any GP study (*w*_*i*_ = 1) using only treatments in *T*_*c*_ (No DW)
    (ii) moderate downweight (i.e., *w*_*i*_ ∼ Beta(3, 3)) to high risk of bias (RoB) GP studies using only treatments in *T*_*c*_ (RoB DW). The risk of bias was assessed according to the Cochrane’s RoB tool (Sterne et al., 2019) and in total 16 studies were rated at high RoB for GP.
    (iii) no downweight for any GP study evaluating treatments in *T*_*c*_ and moderate downweight (i.e., *w*_*i*_ ∼ Beta(3, 3)) for those evaluating at least one treatment in *T*_*a*_ − *T*_*c*_ (NCT DW). This means that here we use the full network of GP with the 34 treatments and we downweight all studies that contain at least one treatment that has never been evaluated in CA (107 studies).
  - prior distributions based on expert opinion for the location parameter combined the three above (i–iii) possibilities for the scale parameters.
3. NMA for CA with non-informative priors (i.e., “standard” NMA).
4. Pairwise meta-analysis for CA with non-informative priors.

All the analyses were conducted using the rjags (22) package through the R statistical software (R version 4.0.3, 2020-10-10) (23). For all models, we ran two chains in parallel, performed 50,000 iterations and discarded the first 10,000 samples of each chain. We checked the chains convergence using the Gelman–Rubin criterion (Brooks & Gelman, 1998) with a value below 1.1 to indicate failure of convergence. We also visually checked the Markov chains using trace plots to inspect the sampling behavior and assess mixing across chains and convergence. The R code and the data used in this article are available in the Appendix and can also be accessed at: https://github.com/TEvrenoglou/codes_sharing_information

### 4.2 Elicitation of expert’s opinion

We prepared a questionnaire and circulated it to psychiatrists with experience in treating schizophrenia for CA and GP (available in Appendix 2). We asked each expert to provide an estimate of the “expected” mean score reduction in the PANSS scale from baseline to endpoint for each drug for CA given the pooled mean score reduction obtained from the data for GP. We further asked them to provide a measure of uncertainty around the given expected mean scores (a) in the form of standard deviation and (b) in a 10-point scale of their confidence. We obtained in total 22 responses which we averaged for each drug using the meta-analysis model given in Equations (7)–(8). We used a non-informative N(0, 10000) and a weakly informative HN(1) prior for the means $\xi_{j}^{P_{1}}$, *j* ∈ *T_c_* and the parameter σ, respectively.

### 4.3 Results

**Figure 2:**
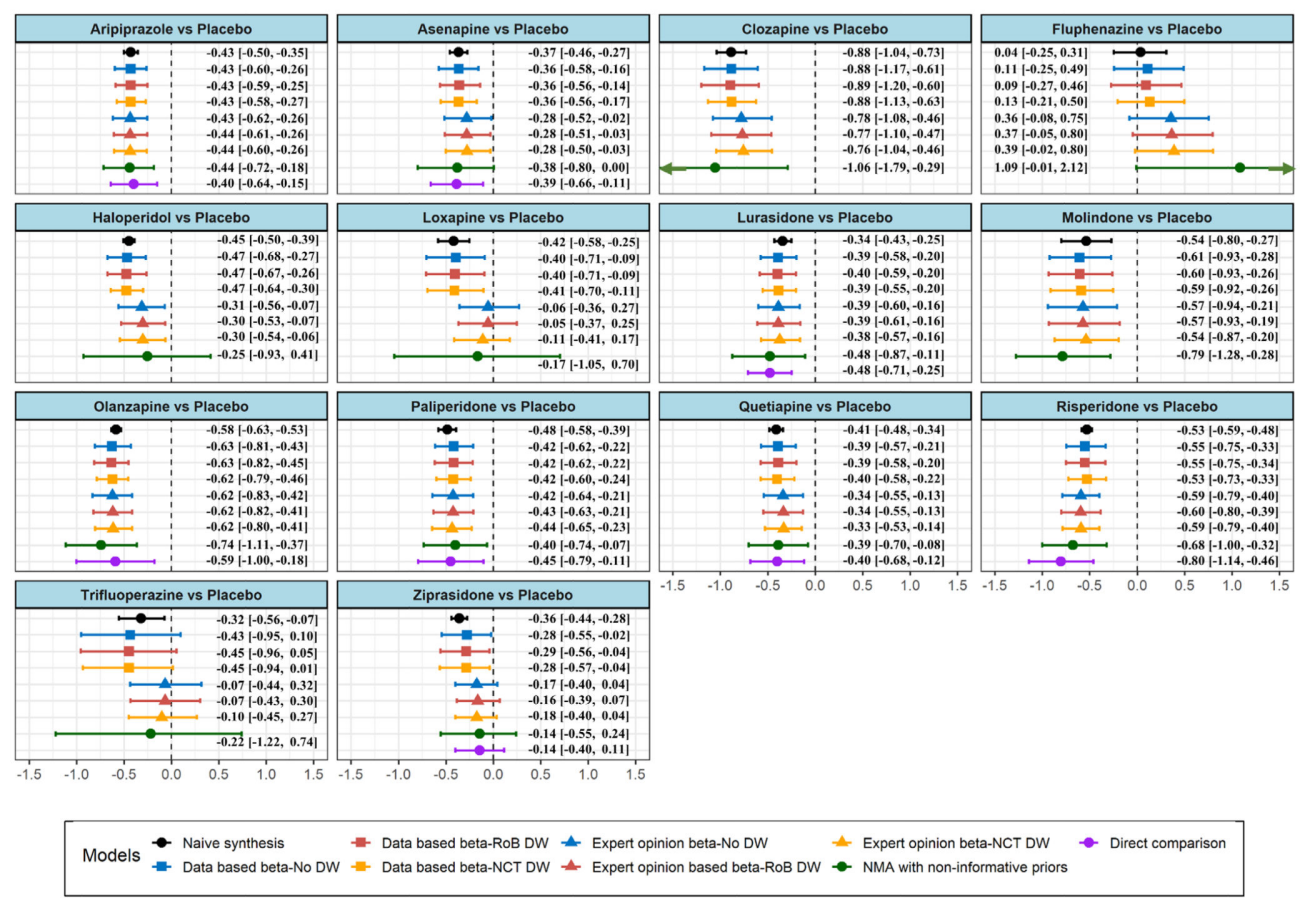
Results from the analysis of the network defined for the population of CA. The outcome of interest is the reduction of the overall schizophrenia symptoms and the effect measure is the standardized mean difference. DW, downweight; RoB, risk of bias; NCT, non-common treatment.

The estimates for the basic comparisons of the CA network are depicted in Figure 2 and for all relative comparisons in Online Appendix 1, Tables 10–17. As expected, for most comparisons, the use of informative priors leads to a substantial improvement in the precision of the relative effects. The naïve synthesis model provides the most precise results but relies on a strong assumption that is likely to be implausible. Interestingly, CA results from the standard NMA model (with non-informative priors) tend to be less precise than the respective direct estimates. This occurs often in sparse networks because indirect comparisons as well as heterogeneity are estimated with large uncertainty. Overall, standard NMA most often does not give any insight for comparisons without direct evidence as it yields very large credible intervals. For some of these comparisons (e.g., Haloperidol vs. Placebo), the NMA models with informative priors result in more conclusive relative effect estimates. In terms of point estimates, results appear generally robust across the different models with only few exceptions, such as the extreme cases of Fluphenazine and Trifluoperazine. Those two are very old drugs for which the evidence is outdated and sparse in both GP and CA networks. The latter implies that the informative prior can dominate in the analysis and yield to estimate closer to the GP. Overall, point estimates appeared to be more robust for the basic comparisons for which direct evidence is available in the network of CA. Finally, no convergence issues were identified across the chains. The corresponding trace plots are available in Appendix 1, Figures 8–13.

Only small differences can be observed among the six models with informative priors, suggesting that the approach of informing the location and the scale parameters does not affect materially the final predictions; this is possibly due to the huge amount of data in the GP network. For a few comparisons, the data-based approach for ***β*** provides to some degree different relative effects than those obtained from the expert opinion approach, implying that the experience from clinical practice does not fully agree with the available data. Those comparisons might need to be prioritized for the design of new trials. Of course, when the full GP network was used and only studies comparing treatments in *T*_*a*_ − *T*_*c*_ were downweighted some extra precision was gained. Such an approach might be more useful when the external network is not as dense as in the present dataset.

Assessment of inconsistency was performed using the node-splitting method (Dias et al., 2010). Inference regarding the presence of inconsistency was based on Bayesian *p*-values, and on the visual inspection of the posterior densities of direct and indirect evidence, Bayesian *p*-values were calculated as 2 × min{*P*, 1 − *P*} where *P* is the probability that the difference between direct and indirect comparisons is positive. Additionally, the different antipsychotics and placebo were ranked across all models using the surface under the cumulative ranking curve (SUCRA) (Salanti et al., 2011) method. Overall, the respective posterior densities of the direct and indirect estimates appeared to be quite similar across the different com-parisons and models and no *p*-value indicated lack of consistency (Online Appendix 1, Figures 2–6, Tables 2–9). In terms of ranking, the results appear to be generally robust across models including the naïve synthesis and the standard NMA model. Clozapine was always ranked as the most effective antipsychotic. Finally, Olanzapine, Risperidone, and Molindone were ranked in second, third, and fourth positions, respectively, in most of the fitted models (Appendix 1, Figure 7).

## 5 Discussion

In this paper, we present a Bayesian framework for NMA of sparse networks aiming to improve the performance of the relative effect estimates in terms of precision and reliability. To this end, we borrow information using external data from a dense network that targets a different subgroup of the population. We model the differences between the two populations with a two-stage approach. At the first stage, we analyze only the dense network, and we extrapolate its results to the sparse network though the incorporation of a location parameter in the distribution of the summary relative effects. Then, we use the extrapolated results to construct informative prior distributions for the target population subgroup. Similar approaches have been used previously at the level of primary studies (Dunne et al., 2011; Dunoyer, 2011; Hampson et al., 2014; Rhodes et al., 2015). We further introduce a scale parameter that inflates the variance of the studies in the dense network to reflect the potential uncertainty regarding the assumed relationship between the two population subgroups (Hlavin et al., 2016; Wadsworth et al., 2018).

We proposed two different approaches for informing the location parameters; one based on the data and one based on expert opinion. The former is easier to implement but the data are used in both stages: once to form the priors and once to perform the NMA. This is a drawback of the data-based approach as it might introduce some correlation that is ignored. In addition, this approach requires that a certain number of direct comparisons are available in both networks; otherwise, the use of non-informative priors would be necessary for several *β*s at the first stage. The prior elicitation approach is general as it does not rely on the amount of direct evidence. However, results from such an approach are always subjective to some degree and handling disagreements between different experts might be challenging. Here, we requested the necessary information from the psychiatrists through a questionnaire and some of them raised concerns that it was difficult for them to make a suggestion. Other ways to elicit information from experts exist. For example, Turner et al. used a three-stage elicitation approach where the experts were interviewed through 1:1 meetings or by telephone (Turner et al., 2022). Alternatively, information on these parameters may be obtained by using, when available, external data, such as from observational studies, electronic health records, or other networks pertaining to populations with similar characteristics.

In our motivating example, the two approaches for constructing priors for the location parameters were generally in agreement except the comparison between Loxapine and Placebo where, in contrast to the data-based approach, the analyses based on the expert opinion may not support the presence of an important effect. In all other comparisons, despite some differences in the magnitude of the estimated summary effects, the two approaches yielded similar conclusions. Regarding the scale parameters, we applied no downweight for trials considered more relevant to our target population and moderate downweight for studies that were considered less relevant or reliable. The results remained robust across the different downweighing schemes that we used. However, the use of Beta(3, 3) prior for the *w*_*i*_s can be considered a conservative choice as it is centered at 0.5 and provides similar mass to both the left and right tails allowing for both strong and mild downweight, for example, *w*_*i*_ ≈ 0 or *w*_*i*_ ≈ 1, respectively. Sensitivity analysis could further consider left-skewed Beta distributions (e.g., Beta(1, 8)) that would assign weights mostly close to 0 and right-skewed Beta^′^s (e.g., Beta(8, 1)) that would assign weights mostly close to 1. Compared to the standard NMA with non-informative priors, our two-stage approach provided substantially more precise estimates. The lack of robustness of the standard NMA model for this dataset is also deduced from the fact that almost always it yielded less precise results than pairwise meta-analysis. As expected, the most precise results were obtained from the naïve synthesis model. Nevertheless, this model does not account for any differences across the two population subgroups.

In the presence of few studies, estimation of the heterogeneity is challenging (Turner et al., 2019). In our analysis, we used a weakly-informative half normal prior distribution for the heterogeneity parameter (Röver et al., 2021). However, a sensitivity analysis on the prior distribution for heterogeneity could be considered as in the absence of sufficient data, the estimation of heterogeneity might be affected by the choice of the prior. For example, using informative priors for heterogeneity could possibly further increase the precision of our approach. Usually, these are based on empirical distributions that depend on the type of outcome and treatment comparisons (Rhodes et al., 2015; Turner et al., 2012, 2015). Alternatively, more sophisticated options that allow the incorporation of external data in the estimation of the heterogeneity parameter may be applied (Turner et al., 2019).

A limitation of our work is that we did not explore under which conditions the introduced location parameters reduce or increase the bias of the summary estimates. This would require to evaluate the proposed Bayesian framework through a simulation study. Nevertheless, data generation in this setting might be challenging as it would require construction of the differences between the two populations based on clinically meaningful baseline characteristics. This merits a lot of further consideration and can form a follow-up article. In addition, such a study could only evaluate the data-based approach for the priors as it would be impossible to obtain expert opinion for many simulated datasets.

Our proposed method was built upon a Bayesian framework as this offers a straightforward and intuitive way to incorporate external information in the analysis through informative prior distributions. Alternatively, a two-stage frequentist framework could be used where the prior information for the location parameters could be incorporated through pseudodata as in Rhodes et al. (Rhodes et al., 2016). However, this approach uses meta-regression to fit an NMA model, and therefore, it is directly applicable only to NMA of two-arm studies (Rhodes et al., 2016). A further possibility may be to model the differences in the two populations through inconsistency parameters using the random inconsistency design-by-treatment interaction model proposed by Jackson et al. (2014). Such an approach would assume different inconsistency parameters for populations *P*_1_ and *P*_2_ but the same for studies within each population. A potential future simulation study could investigate similarities and differences in the estimation between the three approaches.

Overall, the proposed framework offers a reliable approach for assisting the analysis of sparse networks. Of course, its application requires close collaboration between statisticians and experienced clinicians to ensure that sharing of information between different population subgroups is clinically meaningful. Finally, performing NMA in cases of sparse networks will always be a challenging procedure and the best approach will likely be context-dependent.

## Supplementary Material

Supplementary Material

## Data Availability

All data are available in the Supplementary Material and on the following public GitHub repository: https://github.com/TEvrenoglou/codes_sharing_information
