# Supplementary material for: Sharing information across patient subgroups to draw conclusions from sparse treatment networks
Source: Biom J. Author manuscript; Available in PMC 2024 Dec 20. (PMC7617248; doi:10.1002/bimj.202200316)
Supplement: Supplementary Material [file EMS201286-supplement-Supplementary_Material.zip › bimj2569-sup-0002-app2.docx]

# **Appendix 2:** Questionnaire for methodological project on the analysis of psychiatric symptom reduction by antipsychotics in children and adolescents

## **Short introduction to the project (please read this before starting answering the questions)**

## In this project we aim to estimate the effects of different antipsychotics for children and adolescents (Krause et al. 2018^1^). However, the available evidence for this population is very limited. To increase the confidence in our results, we plan to borrow information from available evidence on the same outcome for “general” patients (usually chronic adults with an exacerbation of positive symptoms, for example as those meta-analyzed by Huhn et al. 2019^2^) for whom more evidence is available. Thus, we need to estimate the ‘difference’ between the two populations. To do this properly, we would need your help by answering a questionnaire that we have prepared below.

## More details about our methodology can be found in the following section “**Methodological details related to the project**”.

## Otherwise, please go directly to the questionnaire in the section “**Questions to be answered by experts**” in page 4.

## **Methodological details related to the project**

Sparse data are a common issue in comparative effectiveness research even at the meta-analysis level. We use as an exemplar sparse network, the network formed by 19 RCTs comparing 14 antipsychotics and placebo for the reduction of overall psychiatric symptoms (PANSS total score) in children and adolescents. This network is depicted in **Figure 1**. The 19 RCTs provide 21 out of the 105 (20%) theoretically possible direct comparisons (lines in the graph). Performing a ‘standard’ network meta-analysis (NMA) in this network, which combines direct (example: all studies comparing aripiprazole and paliperidone with each other) and indirect evidence (example: estimating the difference between aripiprazole and paliperidone indirectly from aripiprazole versus placebo and paliperidone versus placebo), is possibly of limited validity. Specifically, the resulting relative effects are equally or even less precise for some comparisons than those obtained from pairwise meta-analysis and the model assumptions cannot be evaluated. However, the pace of new research production for this network is very slow and it is of great importance to use any available piece of information to identify the most beneficial drugs for children and adolescents.


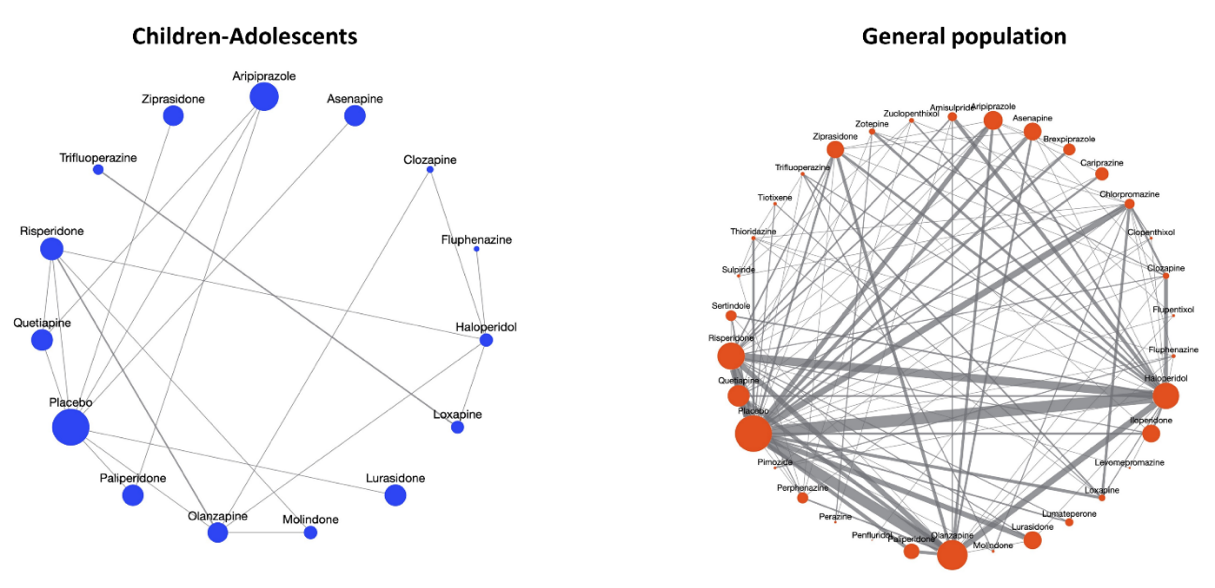
The main idea behind the following questionnaire is that we could use external evidence from a dense (i.e. informative) network after some form of ‘adaptation’ of the results to the population of interest and increase the amount of information for the sparse network for children and adolescents. In other words, we could use this adapted external evidence as ‘prior’ information in a Bayesian NMA model for children and adolescents. In this way, we aim to obtain more precise and more robust (reliable) results for the comparative efficacy of the drugs for children and adolescents.

**Figure 1**


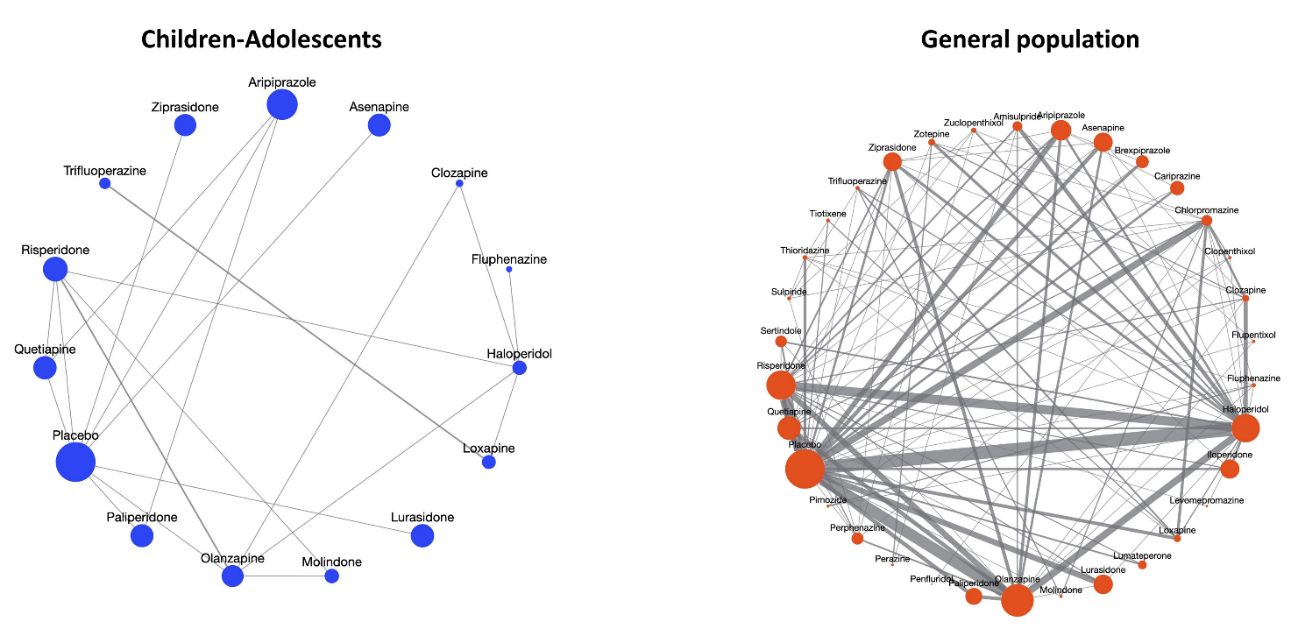
The external informative network we consider here is a network evaluating the drugs for the same outcome (overall symptoms) in “general” adult patients with schizophrenia (**Figure 2**) as they were, for example, meta-analyzed in Huhn et al. 2019^2^. These studies, albeit they do not always use exactly the same definitions, can be characterised as usually chronic patients with an acute exacerbation of positive symptoms. This network consists of 255 studies and 116 direct comparisons. Note that we will only use the evidence from this network for the drugs already existing in the children and adolescent network; hence we will not draw any inference for drugs that have never been used for the population of interest. Of course, some differences among the two populations are inevitable and, therefore, we cannot use the results from the general population without incorporating these differeces. Since we only have aggregate (i.e. summary) data from the studies, we will rely on the average difference between the outcome in the two populations for each drug.

**Figure 2**

From a statistical standpoint, the average difference in the outcome between the two populations is represented by the difference of the mean of the respective distributions. **Figure 3** shows a theoretical example about the difference of the means of the two distributions for the comparison haloperidol vs placebo. Such differences for all comparisons can be obtained either using the data (which we will do, as well) or using expert opinion (this is what we need your help for). Then, these differences will be modelled appropriately to obtain NMA results from the general patient data applicable to children and adolescents which, in turn, will be used as prior information in the NMA for children and adolescents.

**Figure 3**


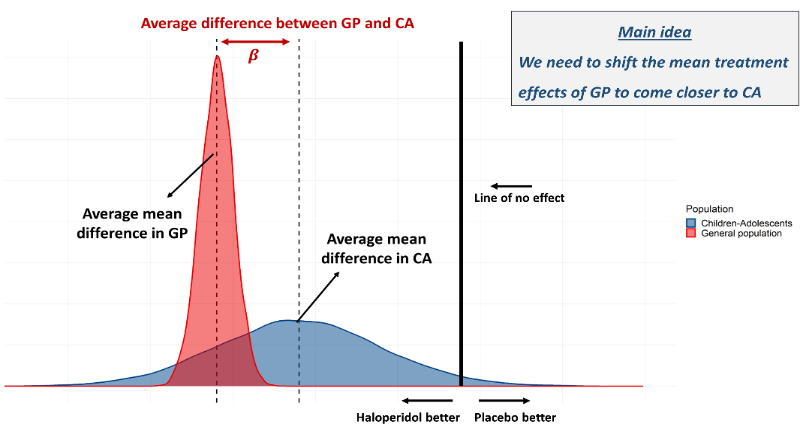


**References**

1. Krause M, Zhu Y, Huhn M, Schneider-Thoma J, Bighelli I, Chaimani A, Leucht S. [Efficacy, acceptability, and tolerability of antipsychotics in children and adolescents with schizophrenia: A network meta-analysis.](https://pubmed.ncbi.nlm.nih.gov/29802039/) Eur Neuropsychopharmacol. 2018 Jun;28(6):659-674

2. Huhn M, Nikolakopoulou A, Schneider-Thoma J, Krause M, Samara M, Peter N, Arndt T, Bäckers L, Rothe P, Cipriani A, Davis J, Salanti G, Leucht S. [Comparative efficacy and tolerability of 32 oral antipsychotics for the acute treatment of adults with multi-episode schizophrenia: a systematic review and network meta-analysis.](https://pubmed.ncbi.nlm.nih.gov/31303314/) Lancet. 2019 Sep 14;394(10202):939-951.

## **Questions to be answered by experts**

**Q1:**  Column 2 of the next table shows a theoretical reduction (change score) in PANSS total score for overall symptoms for the antipsychotics of Figure 2 in “general patients” (chronic adults with acute exacerbation of positive symptoms).

***Please fill into column 3 how much you expect that the corresponding reductions would be for children and adolescents. Please, also provide a standard deviation for the value you provide in column 4.***

You may skip the antipsychotics which you are not familiar with.

***Please also tick the box in column 5 regarding how you think the reduction of the PANSS total score in children and adolescents qualitatively differs from that in general patients.***

**The first row in red presents a fictional example:** If the PANSS total score reduction from baseline to endpoint for “general patients” for a given fictional antipsychotic X is -26 then a possible answer for children and adolescents may be:

- Expected PANSS total score reduction from baseline to endpoint in Children-Adolescents=-29 meaning three points more improvement
- Standard Deviation for the expected PANSS reduction in Children-Adolescents =$\pm10$
- Expected treatment response in qualitative terms in Children-Adolescents compared to “general patients” = A bit better

| **1. Drug** | **2. PANSS total score reduction from baseline to endpoint for “general adult patients” (chronic adults with acute exacerbation of positive symptoms)** | **3. Expected PANSS total score reduction from baseline to endpoint in Children-Adolescents** | **4. Standard deviation for the expected**  **PANSS reduction**  **in Children-Adolescents** | **5. Expected treatment response in qualitative terms in Children-Adolescents compared to “general adult patients”** |
| --- | --- | --- | --- | --- |
| **Fictional antipsychotic**  **X** | -26 | -29 | $\pm10$ | \|  \|  \|  \|  \|  \| \| --- \| --- \| --- \| --- \| --- \| \| **Much better** \| **A bit better** \| **Same** \| **A bit worse** \| **Much worse** \| |
| **Clozapine** | -27.8 |  |  | \|  \|  \|  \|  \|  \| \| --- \| --- \| --- \| --- \| --- \| \| **Much better** \| **A bit better** \| **Same** \| **A bit worse** \| **Much worse** \| |
| **Olanzapine** | -21.2 |  |  | \|  \|  \|  \|  \|  \| \| --- \| --- \| --- \| --- \| --- \| \| **Much better** \| **A bit better** \| **Same** \| **A bit worse** \| **Much worse** \| |
| **Risperidone** | -21 |  |  | \|  \|  \|  \|  \|  \| \| --- \| --- \| --- \| --- \| --- \| \| **Much better** \| **A bit better** \| **Same** \| **A bit worse** \| **Much worse** \| |
| **Paliperidone** | -19.8 |  |  | \|  \|  \|  \|  \|  \| \| --- \| --- \| --- \| --- \| --- \| \| **Much better** \| **A bit better** \| **Same** \| **A bit worse** \| **Much worse** \| |
| **Haloperidol** | -19.4 |  |  | \|  \|  \|  \|  \|  \| \| --- \| --- \| --- \| --- \| --- \| \| **Much better** \| **A bit better** \| **Same** \| **A bit worse** \| **Much worse** \| |
| **Loxapine** | -19 |  |  | \|  \|  \|  \|  \|  \| \| --- \| --- \| --- \| --- \| --- \| \| **Much better** \| **A bit better** \| **Same** \| **A bit worse** \| **Much worse** \| |
| **Quetiapine** | -18.4 |  |  | \|  \|  \|  \|  \|  \| \| --- \| --- \| --- \| --- \| --- \| \| **Much better** \| **A bit better** \| **Same** \| **A bit worse** \| **Much worse** \| |
| **Molindone** | -18.4 |  |  | \|  \|  \|  \|  \|  \| \| --- \| --- \| --- \| --- \| --- \| \| **Much better** \| **A bit better** \| **Same** \| **A bit worse** \| **Much worse** \| |
| **Aripiprazole** | -18.2 |  |  | \|  \|  \|  \|  \|  \| \| --- \| --- \| --- \| --- \| --- \| \| **Much better** \| **A bit better** \| **Same** \| **A bit worse** \| **Much worse** \| |
| **Ziprasidone** | -18.2 |  |  | \|  \|  \|  \|  \|  \| \| --- \| --- \| --- \| --- \| --- \| \| **Much better** \| **A bit better** \| **Same** \| **A bit worse** \| **Much worse** \| |
| **Asenapine** | -17.8 |  |  | \|  \|  \|  \|  \|  \| \| --- \| --- \| --- \| --- \| --- \| \| **Much better** \| **A bit better** \| **Same** \| **A bit worse** \| **Much worse** \| |
| **Lurasidone** | -17.2 |  |  | \|  \|  \|  \|  \|  \| \| --- \| --- \| --- \| --- \| --- \| \| **Much better** \| **A bit better** \| **Same** \| **A bit worse** \| **Much worse** \| |
| **Fluphenazine** | -14.8 |  |  | \|  \|  \|  \|  \|  \| \| --- \| --- \| --- \| --- \| --- \| \| **Much better** \| **A bit better** \| **Same** \| **A bit worse** \| **Much worse** \| |
| **Trifluoperazine** | -14.8 |  |  | \|  \|  \|  \|  \|  \| \| --- \| --- \| --- \| --- \| --- \| \| **Much better** \| **A bit better** \| **Same** \| **A bit worse** \| **Much worse** \| |
| **Placebo** | -10 |  |  | \|  \|  \|  \|  \|  \| \| --- \| --- \| --- \| --- \| --- \| \| **Much better** \| **A bit better** \| **Same** \| **A bit worse** \| **Much worse** \| |

**Q2:** In a scale from 1 to 10 how confident you are in the numbers that you gave in **Q1**? (1=not confident, 10=very confident)

| **1** | **2** | **3** | **4** | **5** | **6** | **7** | **8** | **9** | **10** |
| --- | --- | --- | --- | --- | --- | --- | --- | --- | --- |
|  |  |  |  |  |  |  |  |  |  |

**Q3:** What are the most probable population related factors that may differentiate the effectiveness of the antipsychotics between general patients and children and adolescents?

|  |
| --- |

**Q4:** Consider that a new drug with very positive results for the general population has come out

(very efficacious without important side effects) but not tested in children and adolescents. Would you consider it for treating children/adolescents with schizophrenia? If yes, in which situations (please fill-in the box below)? **Please disregard any legal concerns with off-label use.**

| Certainly yes |  |
| --- | --- |
| Possibly yes |  |
| Maybe |  |
| Possibly no |  |
| Certainly no |  |

**If not, why would you not consider giving the potential new drug to children and adolescents when evidence is available only for the general population?**

|  |
| --- |

**Q5:** Your main work is

| Research |  |
| --- | --- |
| Clinical practice |  |
| Both |  |

|  |
| --- |

**Q6:** How many years have you been working in the field of schizophrenia?

**Q7:** You have experience with

| Child psychiatry |  |
| --- | --- |
| Adult psychiatry |  |
| Both |  |

**Q8:** What kind of experience do you have with studies about antipsychotics?

| Involved in randomized clinical trials |  |
| --- | --- |
| Involved in meta-analyses of clinical trials |  |
| Involved in observational studies |  |
| Any other study related experience |  |
