# Supplementary material for: Sharing information across patient subgroups to draw conclusions from sparse treatment networks
Source: Biom J. Author manuscript; Available in PMC 2024 Dec 20. (PMC7617248; doi:10.1002/bimj.202200316)
Supplement: Supplementary Material [file EMS201286-supplement-Supplementary_Material.zip › bimj2569-sup-0001-app1.docx]

# **Appendix 1**

## Analyzing the two networks using the community detection algorithm

A formal way to describe the differences between the amount of information in the two networks is the visualization approach proposed by Law et al^1^. This approach uses the network analysis community detection algorithm to determine which parts of the network are well identified. To achieve this, the method is based on an adjacency matrix created using as thresholds the quantiles of the standard errors of the NMA estimates. In their manuscript Law et al^1^. suggested to set multiple thresholds according to the 20%, 40%, 60% and 80% quantiles of the treatment effect standard errors. These quantiles for the networks of GP and CA are shown in **Appendix** **Table 1**. More information about the method can be found in the original publication^1^.

**Appendix Table 1:** The 20%, 40%, 60% and 80% quantile of the standard errors of all the NMA estimates in the networks of General patients and Children-Adolescents.

| **Network of interest** | **20% quantile** | **40% quantile** | **60% quantile** | **80% quantile** |
| --- | --- | --- | --- | --- |
| **GP** | 0.04 | 0.05 | 0.12 | 0.17 |
| **CA** | 0.18 | 0.26 | 0.37 | 0.46 |

Since here we are not only interested to investigate each network but also to compare them with each other, we need to set the threshold according to the quantiles that result into similar or identical standard errors for each network. To conduct the analysis and visualize the results we chose the 80% and 20% quantile in the network of GP and CA, respectively. These correspond to a standard error threshold of 0.17 for GP and 0.18 for CA. The results of the network analysis are shown in **Appendix** **Figure 1**, panel (A) for GP and panel (B) for CA. The nodes in the networks represent the treatments while the connections through the edges indicate that the respective part of the network is well identified. Treatments which are disconnected and far from the network are the parts of the network which are not well identified in terms of the pre-specified threshold. In panel (A) we can observe that almost all the parts of the GP network are very close to each other and thus very well identified. The only part of the network that appears to be far is related to the treatment Molindone. The results for CA in panel (B) reveal the challenges with this sparse network as 40% of the total nodes appear to be not well identified.


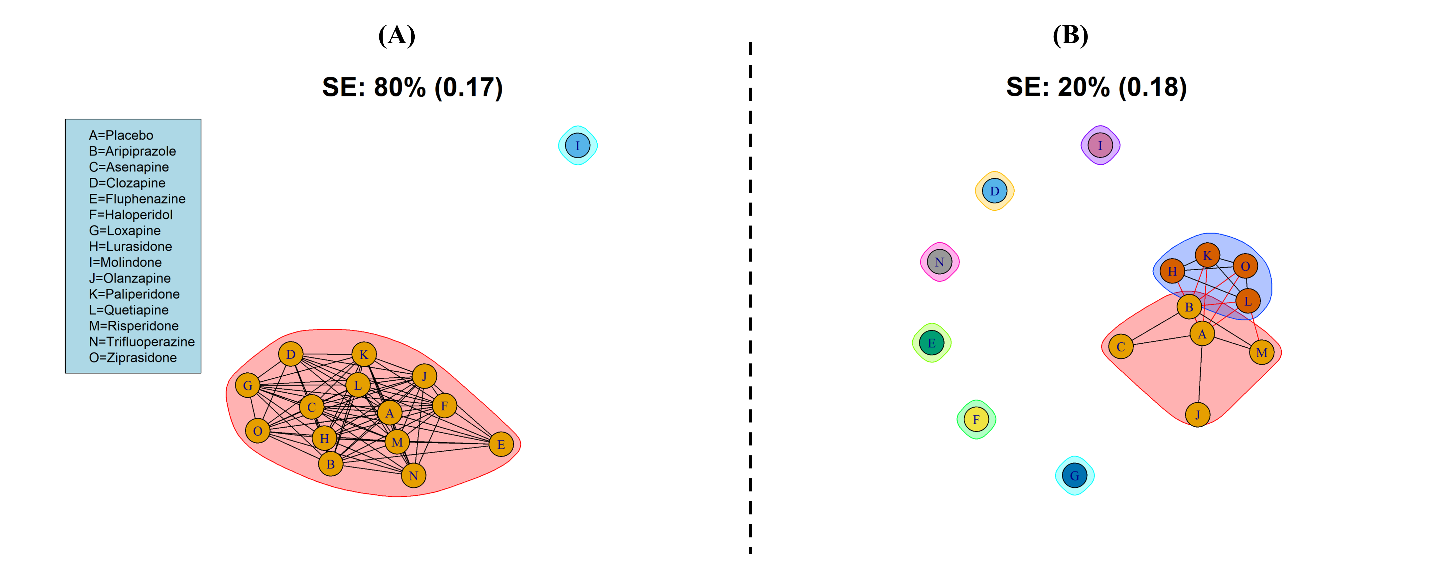


**Appendix Figure 1:** Network analysis results for the GP network in panel (A) and CA network in panel (B). The thresholds for the standard errors in the two networks are 0.17 and 0.18 for GP and CA, respectively. These correspond to the 80% quantile of the standard errors in GP and the 20% quantile of the standard errors in CA. The nodes in the networks represent the different treatments while the edges indicate connections and thus well identified treatments. Isolated treatments which are disconnected from the network indicate the parts of the networks which are not well identified.

## Consistency checks

**Appendix Table 2:** Consistency checks for the naive synthesis model.

| **Comparison** | **Direct [95% CI]** | **Indirect [95% CI]** | **Difference [95% CI]** | **P(diff>0)** | **p-value** |
| --- | --- | --- | --- | --- | --- |
| Aripiprazole vs Placebo | -0.40 [-0.51, -0.28] | -0.45 [-0.54, -0.36] | 0.05 [-0.08, 0.19] | 0.79 | 0.43 |
| Olanzapine vs Placebo | -0.50 [-0.58, -0.42] | -0.59 [-0.66, -0.53] | 0.09 [-0.01, 0.20] | 0.95 | 0.10 |
| Paliperidone vs Placebo | -0.52 [-0.65, -0.40] | -0.54 [-0.67, -0.40] | 0.01 [-0.18, 0.21] | 0.55 | 0.91 |
| Quetiapine vs Placebo | -0.27 [-0.38, -0.16] | -0.40 [-0.50, -0.31] | 0.14 [-0.01, 0.28] | 0.97 | 0.06 |
| Risperidone vs Placebo | -0.37 [-0.45, -0.29] | -0.51 [-0.59, -0.42] | 0.14 [0.02, 0.26] | 0.99 | 0.02 |

**Appendix Table 3:** Consistency checks for the NMA model with informative priors obtained from GP using a data based approach for $\beta$ and no downweight.

| **Comparison** | **Direct [95% CI]** | **Indirect [95% CI]** | **Difference [95% CI]** | **P(diff>0)** | **p-value** |
| --- | --- | --- | --- | --- | --- |
| Aripiprazole vs Placebo | -0.40 [-0.49, -0.31] | -0.50 [-0.80, -0.22] | 0.10 [-0.20, 0.41] | 0.76 | 0.48 |
| Olanzapine vs Placebo | -0.58 [-0.66, -0.51] | -0.69 [-1.02, -0.36] | 0.11 [-0.24, 0.45] | 0.75 | 0.50 |
| Paliperidone vs Placebo | -0.45 [-0.60, -0.30] | -0.34 [-0.71, 0.03] | -0.11 [-0.51, 0.30] | 0.26 | 0.53 |
| Quetiapine vs Placebo | -0.42 [-0.58, -0.26] | -0.32 [-0.73, 0.08] | -0.10 [-0.55, 0.34] | 0.32 | 0.64 |
| Risperidone vs Placebo | -0.50 [-0.68, -0.30] | -0.47 [-0.84, -0.12] | -0.03 [-0.44, 0.39] | 0.45 | 0.89 |

**Appendix Table 4:** Consistency checks for the NMA model with informative priors obtained from GP using a data based approach for $\beta$ and moderate downweight to all GP studies with high RoB.

| **Comparison** | **Direct [95% CI]** | **Indirect [95% CI]** | **Difference [95% CI]** | **P(diff>0)** | **p-value** |
| --- | --- | --- | --- | --- | --- |
| Aripiprazole vs Placebo | -0.40 [-0.50, -0.31] | -0.50 [-0.80, -0.21] | 0.10 [-0.21, 0.40] | 0.75 | 0.51 |
| Olanzapine vs Placebo | -0.59 [-0.66, -0.51] | -0.70 [-1.01, -0.38] | 0.11 [-0.21, 0.44] | 0.77 | 0.46 |
| Paliperidone vs Placebo | -0.45 [-0.59, -0.30] | -0.35 [-0.71, 0.00] | -0.10 [-0.47, 0.29] | 0.31 | 0.62 |
| Quetiapine vs Placebo | -0.42 [-0.57, -0.26] | -0.32 [-0.72, 0.10] | -0.10 [-0.53, 0.35] | 0.32 | 0.63 |
| Risperidone vs Placebo | -0.51 [-0.70, -0.31] | -0.47 [-0.80, -0.12] | -0.04 [-0.44, 0.36] | 0.42 | 0.85 |

**Appendix Table 5:** Consistency checks for the NMA model with informative priors obtained from GP using a data based approach for $\beta$ and moderate downweight to all GP studies with interventions in $T_{a}-T_{c}$.

| **Comparison** | **Direct [95% CI]** | **Indirect [95% CI]** | **Difference [95% CI]** | **P(diff>0)** | **p-value** |
| --- | --- | --- | --- | --- | --- |
| Aripiprazole vs Placebo | -0.41 [-0.50, -0.32] | -0.51 [-0.82, -0.22] | 0.10 [-0.20, 0.41] | 0.74 | 0.53 |
| Olanzapine vs Placebo | -0.59 [-0.66, -0.51] | -0.68 [-1.02, -0.36] | 0.10 [-0.25, 0.44] | 0.72 | 0.56 |
| Paliperidone vs Placebo | -0.45 [-0.59, -0.32] | -0.34 [-0.70, 0.03] | -0.11 [-0.49, 0.26] | 0.28 | 0.55 |
| Quetiapine vs Placebo | -0.42 [-0.57, -0.27] | -0.33 [-0.70, 0.07] | -0.10 [-0.52, 0.31] | 0.33 | 0.66 |
| Risperidone vs Placebo | -0.48 [-0.65, -0.31] | -0.48 [-0.83, -0.12] | 0.00 [-0.38, 0.39] | 0.50 | 0.99 |

**Appendix Table 6:** Consistency checks for the NMA model with informative priors obtained from GP using expert’s opinion for $\beta$ and no downweight.

| **Comparison** | **Direct [95% CI]** | **Indirect [95% CI]** | **Difference [95% CI]** | **P(diff>0)** | **p-value** |
| --- | --- | --- | --- | --- | --- |
| Aripiprazole vs Placebo | -0.43 [-0.60, -0.25] | -0.47 [-0.76, -0.18] | 0.05 [-0.30, 0.38] | 0.62 | 0.77 |
| Olanzapine vs Placebo | -0.59 [-0.79, -0.39] | -0.67 [-0.99, -0.36] | 0.08 [-0.28, 0.47] | 0.67 | 0.67 |
| Paliperidone vs Placebo | -0.47 [-0.68, -0.26] | -0.35 [-0.72, 0.01] | -0.12 [-0.54, 0.33] | 0.28 | 0.56 |
| Quetiapine vs Placebo | -0.31 [-0.49, -0.12] | -0.37 [-0.76, 0.02] | 0.06 [-0.40, 0.50] | 0.62 | 0.77 |
| Risperidone vs Placebo | -0.63 [-0.82, -0.43] | -0.42 [-0.78, -0.05] | -0.21 [-0.62, 0.21] | 0.17 | 0.33 |

**Appendix Table 7:** Consistency checks for the NMA model with informative priors obtained from GP using expert’s opinion for $\beta$ and moderate downweight to all GP studies with high RoB.

| **Comparison** | **Direct [95% CI]** | **Indirect [95% CI]** | **Difference [95% CI]** | **P(diff>0)** | **p-value** |
| --- | --- | --- | --- | --- | --- |
| Aripiprazole vs Placebo | -0.42 [-0.60, -0.25] | -0.47 [-0.76, -0.16] | 0.05 [-0.31, 0.40] | 0.63 | 0.74 |
| Olanzapine vs Placebo | -0.58 [-0.77, -0.40] | -0.68 [-1.02, -0.36] | 0.10 [-0.29, 0.49] | 0.69 | 0.63 |
| Paliperidone vs Placebo | -0.48 [-0.69, -0.26] | -0.35 [-0.71, 0.04] | -0.13 [-0.59, 0.30] | 0.27 | 0.55 |
| Quetiapine vs Placebo | -0.31 [-0.50, -0.12] | -0.36 [-0.76, 0.05] | 0.05 [-0.39, 0.50] | 0.58 | 0.84 |
| Risperidone vs Placebo | -0.62 [-0.82, -0.43] | -0.42 [-0.78, -0.04] | -0.20 [-0.61, 0.20] | 0.17 | 0.34 |

**Appendix Table 8:** Consistency checks for the NMA model with informative priors obtained from GP using expert’s opinion for $\beta$ and moderate downweight to all GP studies with interventions in $T_{a}-T_{c}$.

| **Comparison** | **Direct [95% CI]** | **Indirect [95% CI]** | **Difference [95% CI]** | **P(diff>0)** | **p-value** |
| --- | --- | --- | --- | --- | --- |
| Aripiprazole vs Placebo | -0.42 [-0.60, -0.24] | -0.48 [-0.76, -0.18] | 0.05 [-0.30, 0.39] | 0.63 | 0.74 |
| Olanzapine vs Placebo | -0.58 [-0.77, -0.39] | -0.65 [-0.99, -0.35] | 0.07 [-0.31, 0.47] | 0.65 | 0.71 |
| Paliperidone vs Placebo | -0.48 [-0.70, -0.29] | -0.35 [-0.74, 0.02] | -0.13 [-0.55, 0.29] | 0.26 | 0.52 |
| Quetiapine vs Placebo | -0.31 [-0.50, -0.12] | -0.37 [-0.74, 0.04] | 0.06 [-0.38, 0.48] | 0.62 | 0.76 |
| Risperidone vs Placebo | -0.62 [-0.82, -0.44] | -0.41 [-0.77, -0.06] | -0.21 [-0.62, 0.19] | 0.14 | 0.28 |

**Appendix Table 9:** Consistency checks for the NMA model with non-informative priors.

| **Comparison** | **Direct [95% CI]** | **Indirect [95% CI]** | **Difference [95% CI]** | **P(diff>0)** | **p-value** |
| --- | --- | --- | --- | --- | --- |
| Aripiprazole vs Placebo | -0.40 [-0.88, 0.07] | -0.49 [-0.96, 0.07] | 0.09 [-0.64, 0.77] | 0.64 | 0.72 |
| Olanzapine vs Placebo | -0.59 [-1.06, -0.09] | -0.96 [-1.53, -0.44] | 0.38 [-0.35, 1.12] | 0.85 | 0.31 |
| Paliperidone vs Placebo | -0.43 [-0.91, 0.10] | -0.35 [-0.93, 0.25] | -0.08 [-0.86, 0.71] | 0.4 | 0.81 |
| Quetiapine vs Placebo | -0.40 [-0.93, 0.09] | -0.38 [-0.88, 0.14] | -0.03 [-0.78, 0.67] | 0.47 | 0.95 |
| Risperidone vs Placebo | -0.80 [-1.23, -0.37] | -0.48 [-0.99, 0.06] | -0.33 [-1.00, 0.36] | 0.17 | 0.34 |


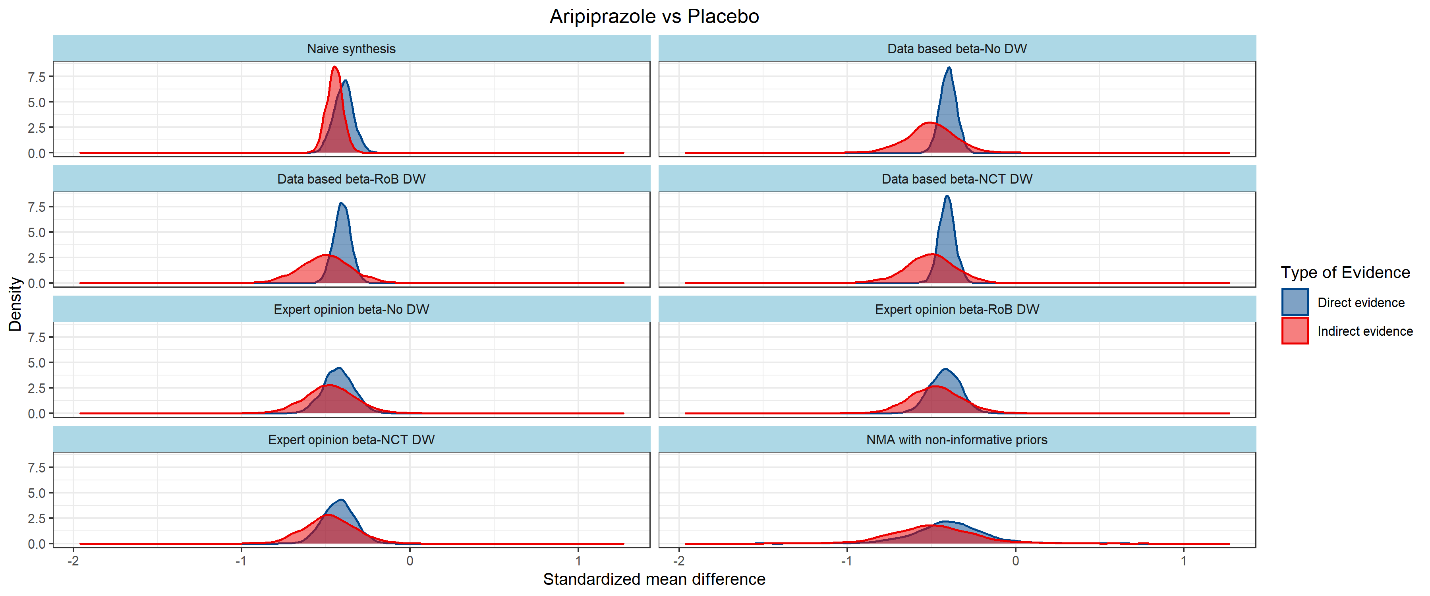


**Appendix Figure 2:** Posterior densities for direct and indirect evidence in terms of comparison Aripiprazole versus Placebo across all the different models.


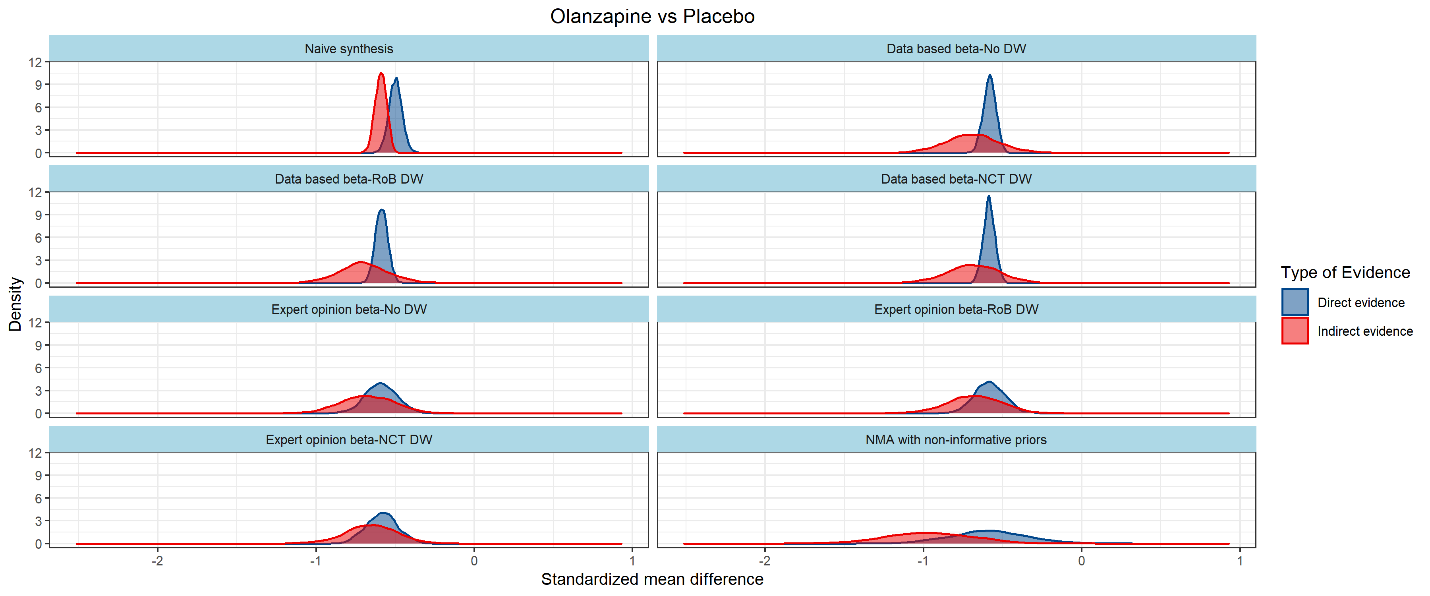


**Appendix Figure 3:** Posterior densities for direct and indirect evidence in terms of comparison Olanzapine versus Placebo across all the different models.


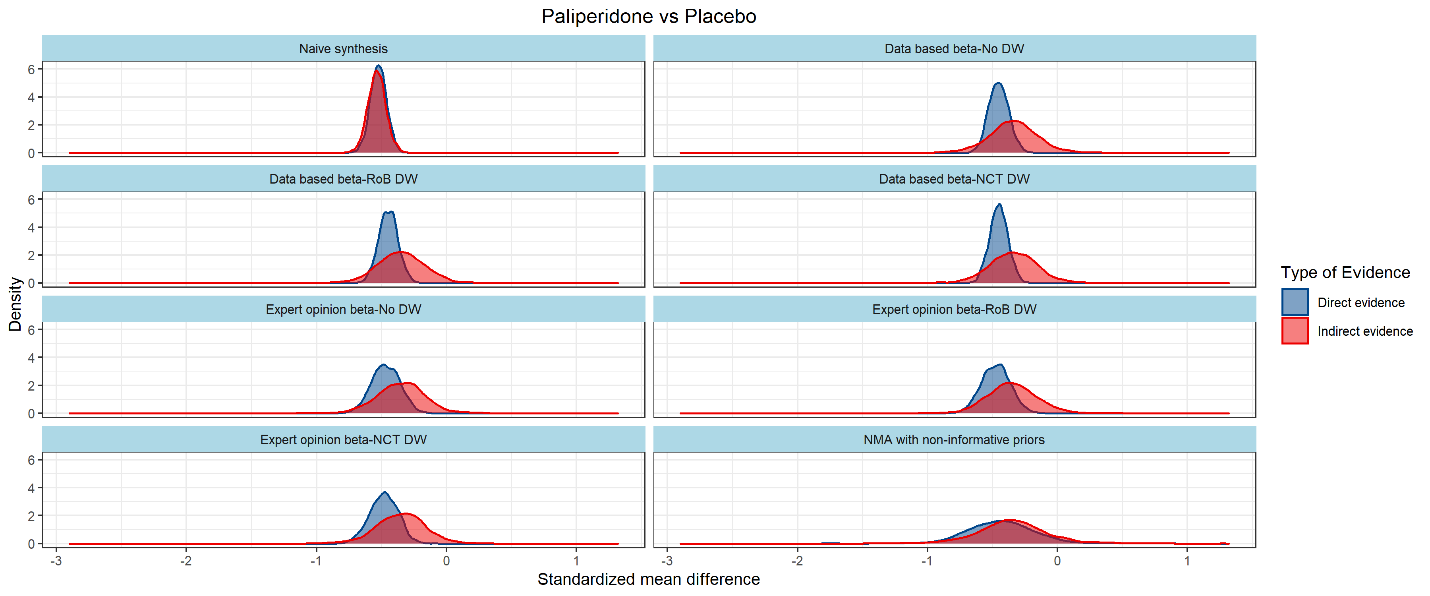


**Appendix Figure 4:** Posterior densities for direct and indirect evidence in terms of comparison Paliperidone versus Placebo across all the different models.


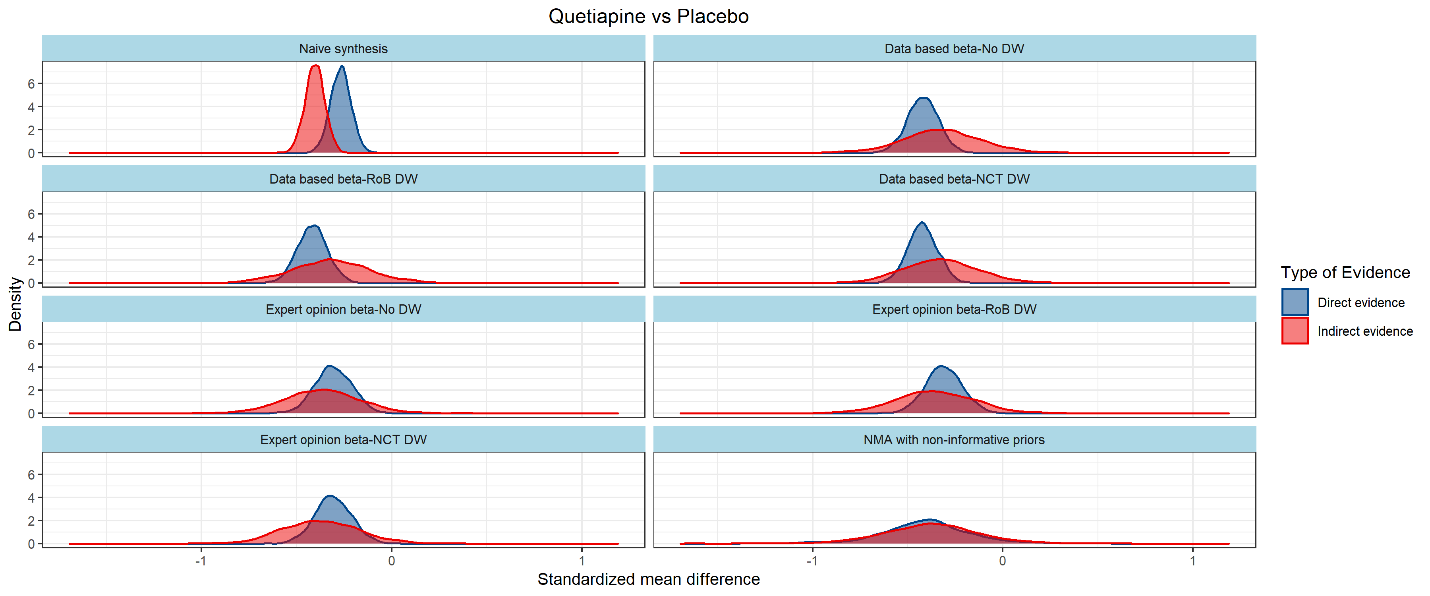


**Appendix Figure 5:** Posterior densities for direct and indirect evidence in terms of comparison Quetiapine versus Placebo across all the different models.


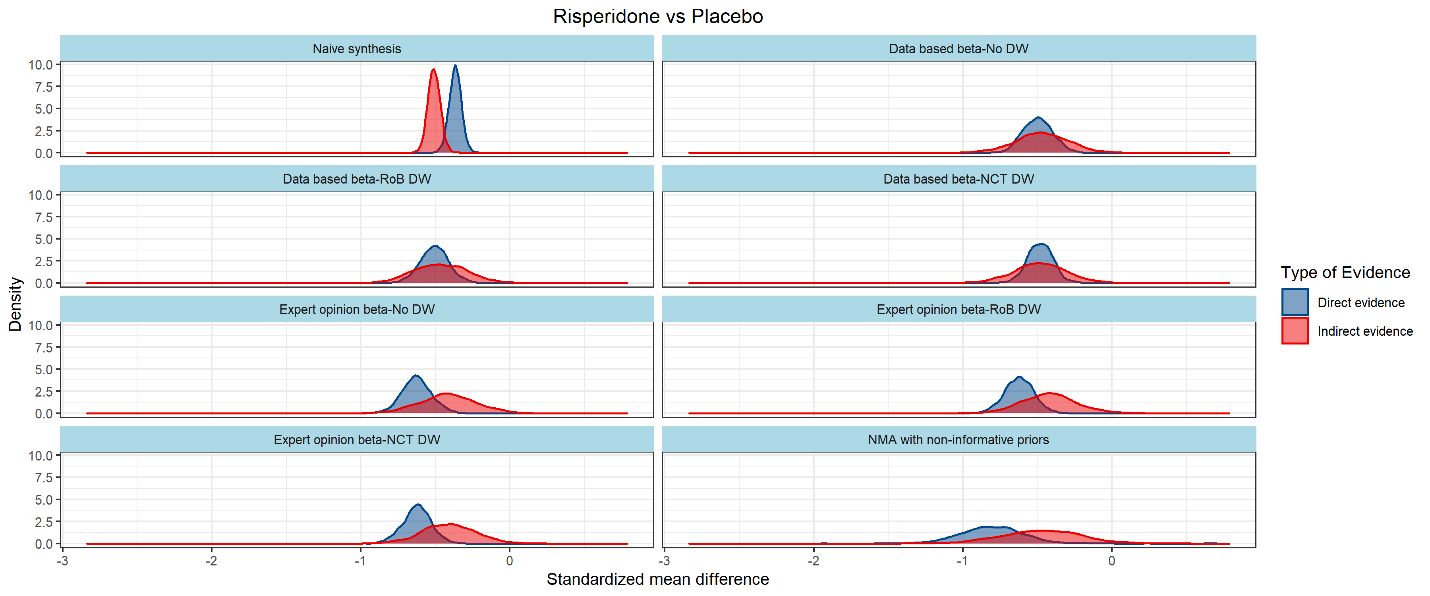


**Appendix Figure 6:** Posterior densities for direct and indirect evidence in terms of comparison Risperidone versus Placebo across all the different models.

## Treatment ranking across all models


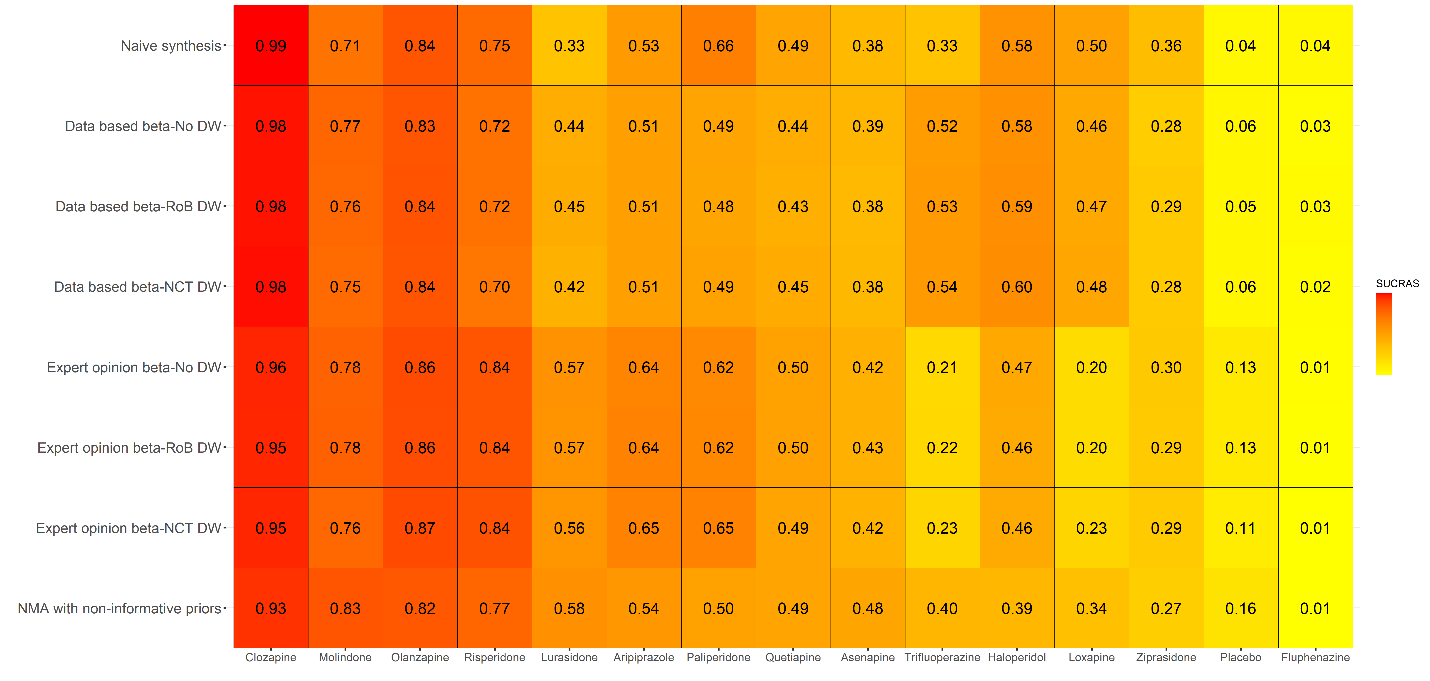


**Appendix Figure 7:** Ranking between all the drugs (14 antipsychotics and Placebo) as obtained from each one of the different models.

## Traceplots


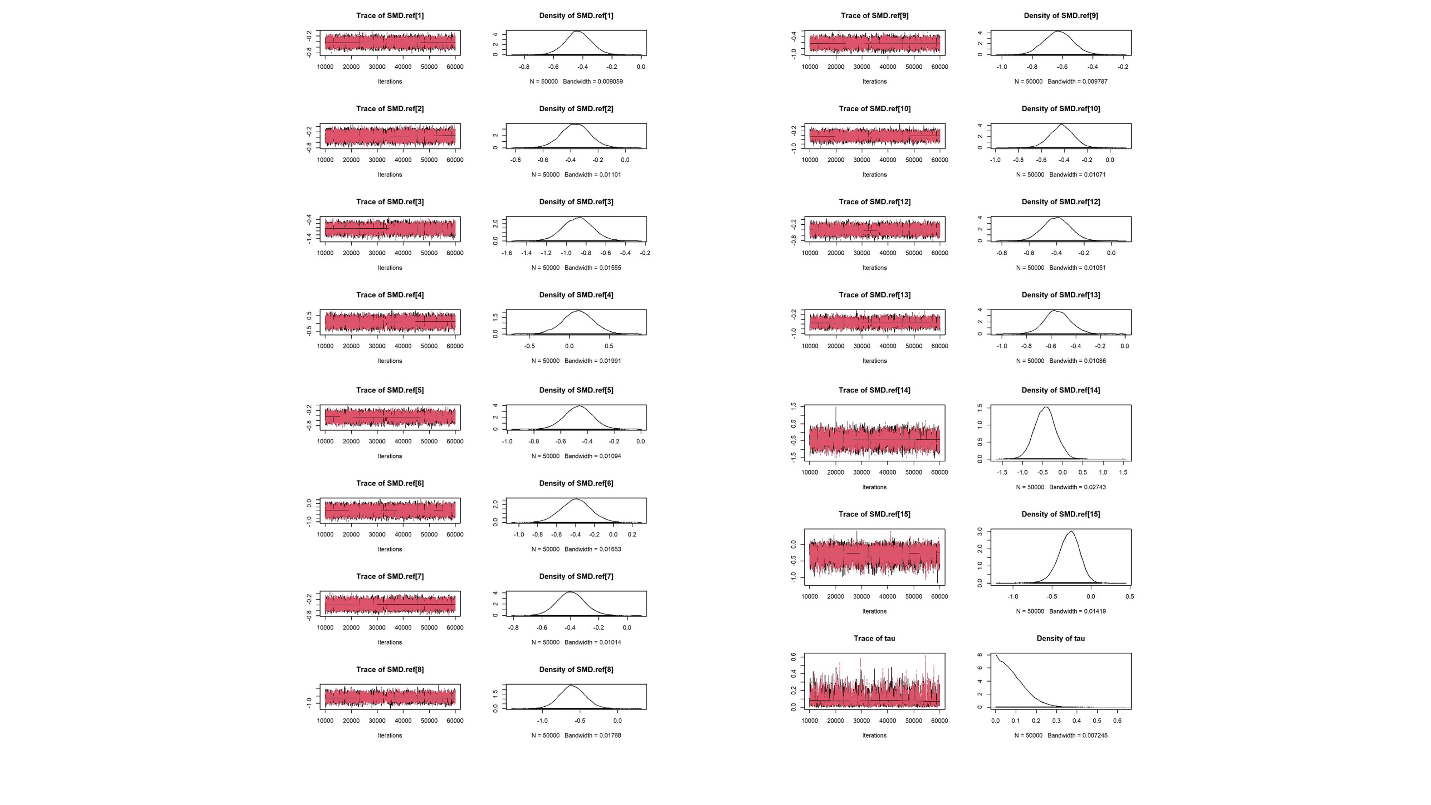


**Appendix Figure 8:** Trace plots for the NMA model with informative priors obtained from GP using a data based approach for $\beta$ and no downweight.


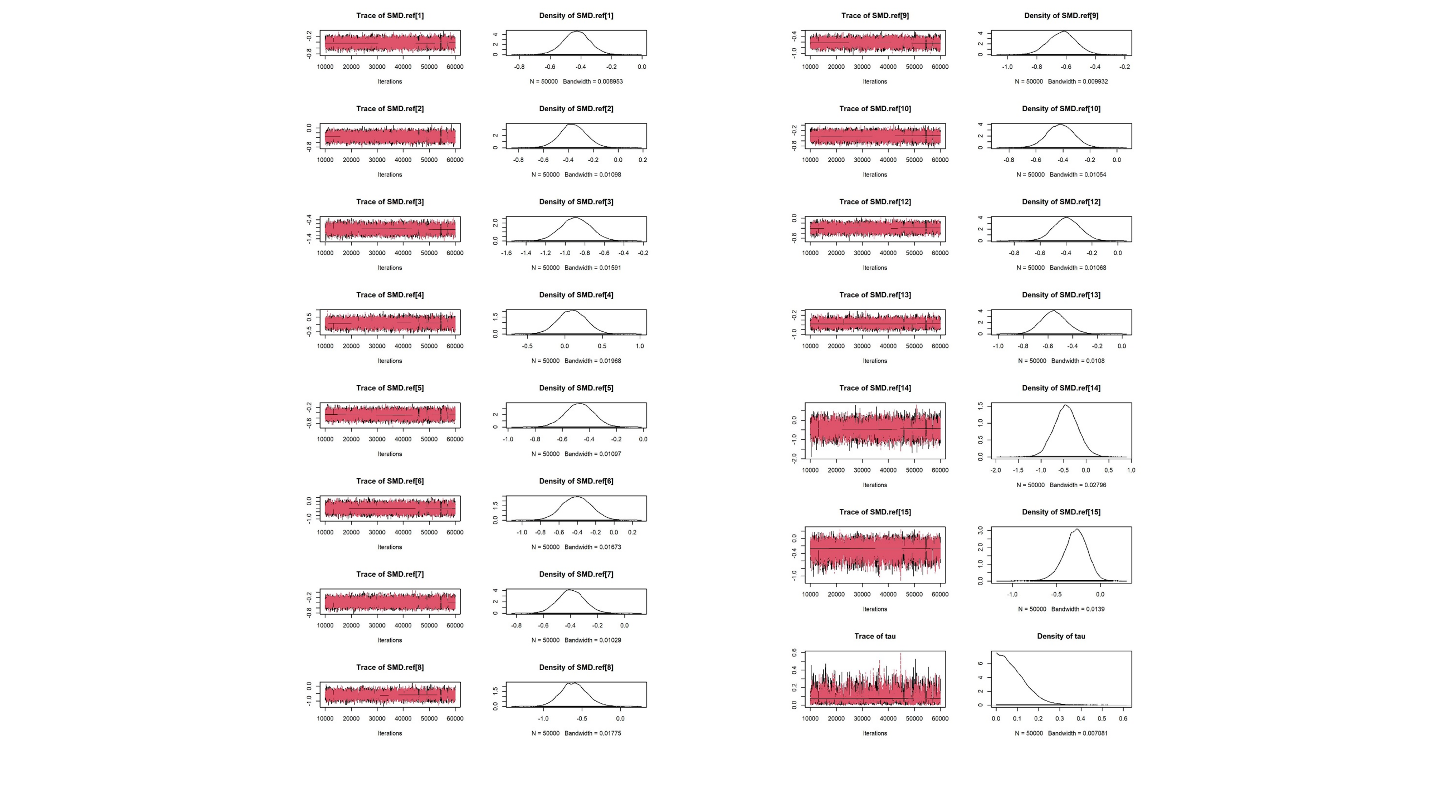


**Appendix Figure 9:** Trace plots for the NMA model with informative priors obtained from GP using a data based approach for $\beta$ and moderate downweight to all GP studies with high RoB


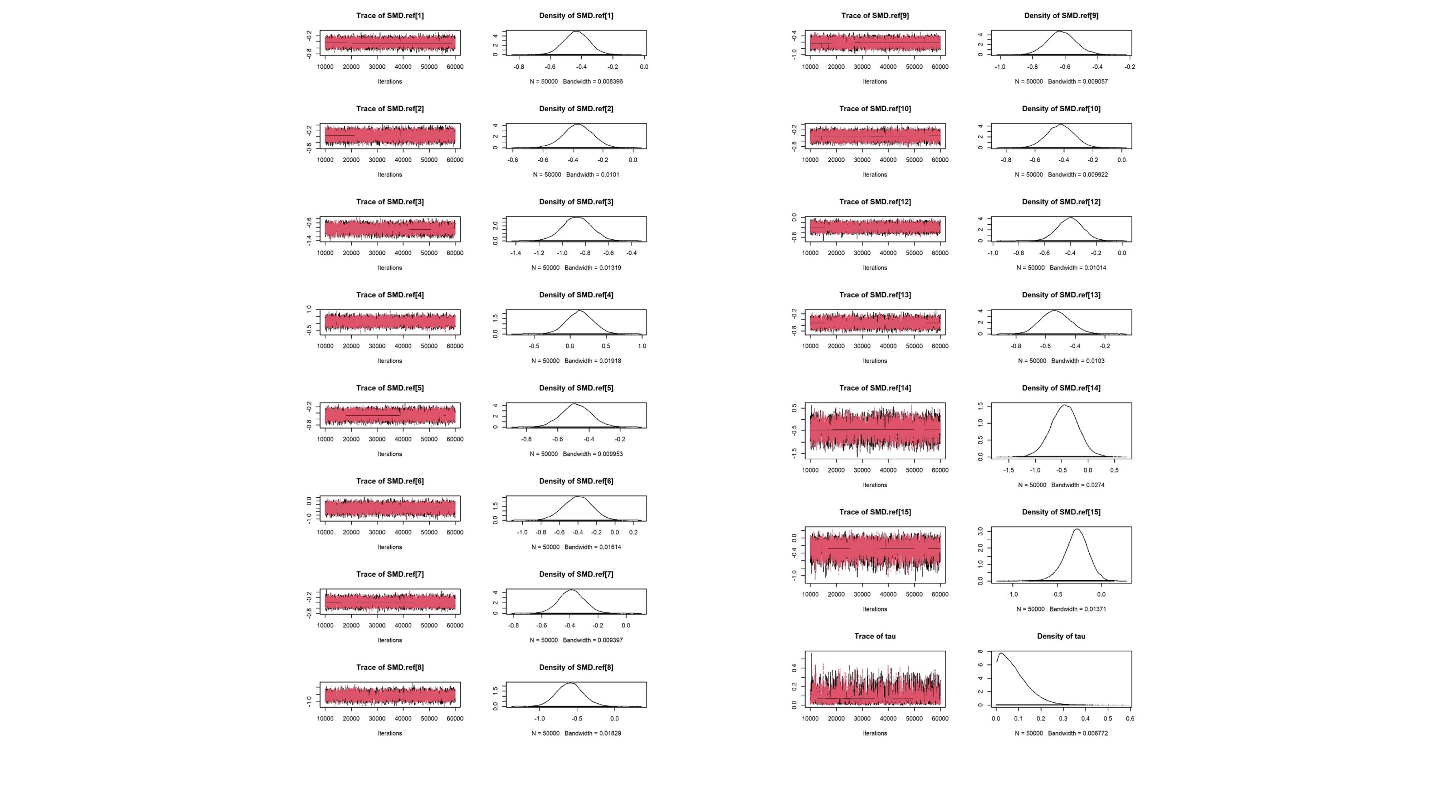


**Appendix Figure 10:** Trace plots for the NMA model with informative priors obtained from GP using a data based approach for $\beta$ and moderate downweight to all GP studies with interventions in $T_{a}$-$T_{c}$


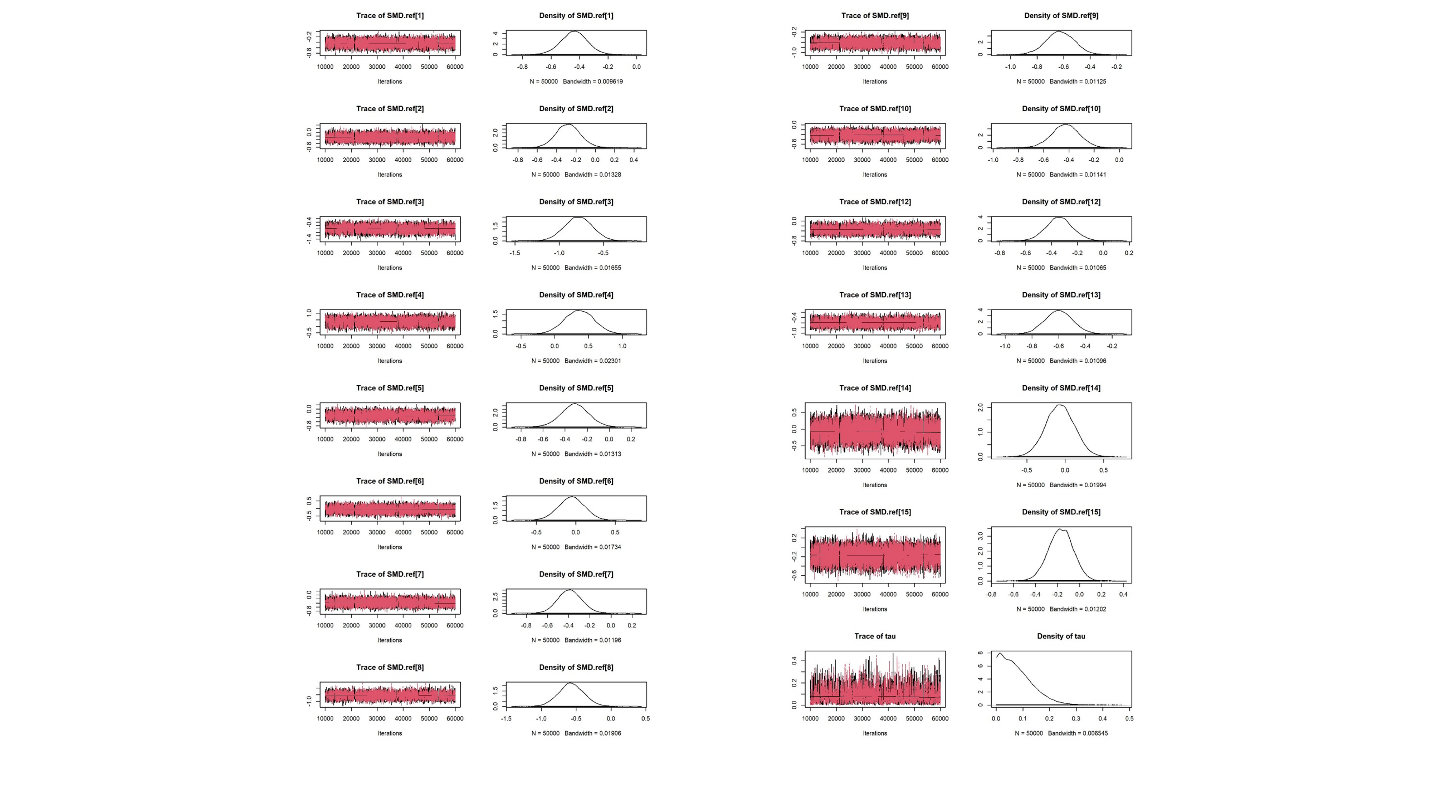


**Appendix Figure 11:** Trace plots for the NMA model with informative priors obtained from GP expert’s opinion for $\beta$ and no downweight.


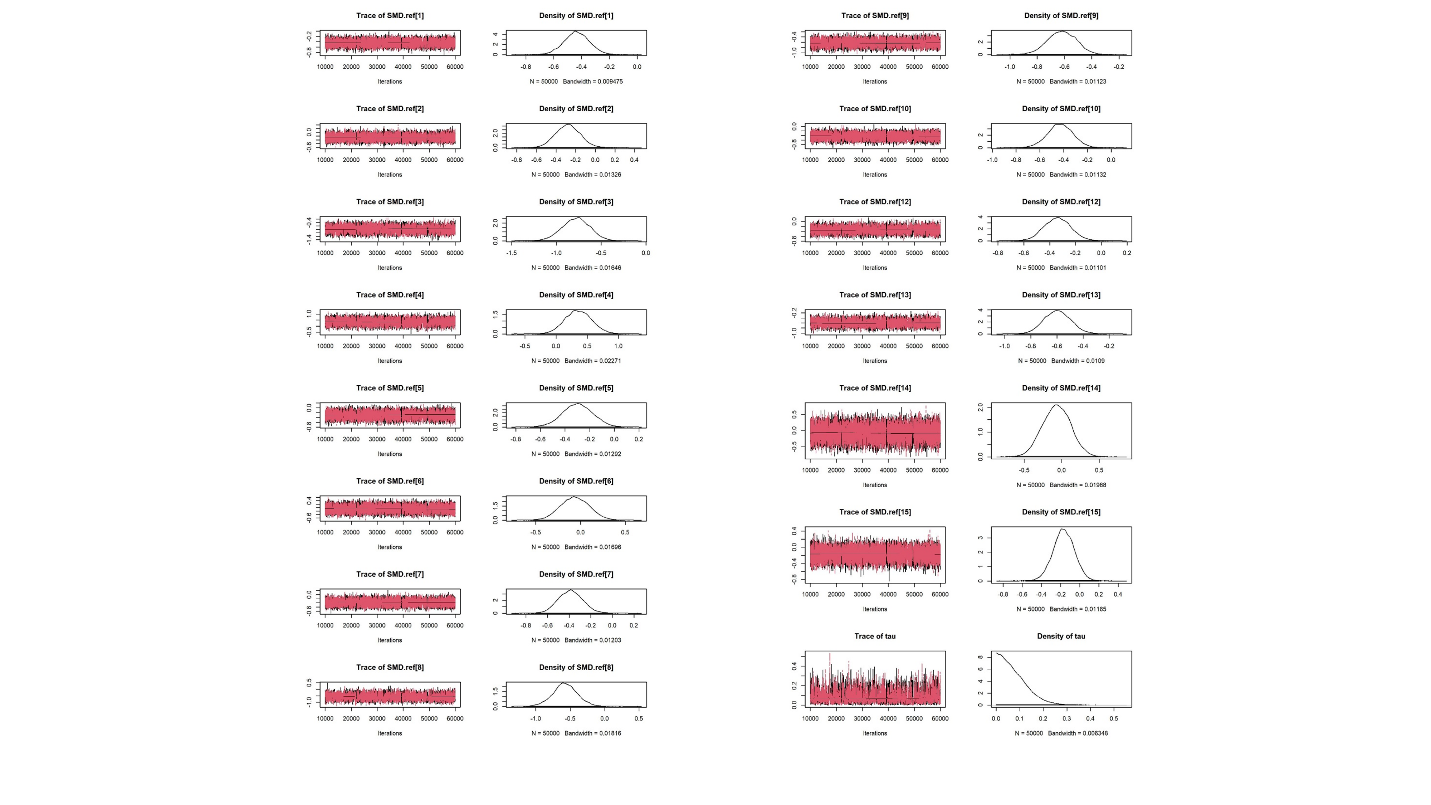


**Appendix Figure 12:** Trace plots for the NMA model with informative priors obtained from GP expert’s opinion for $\beta$ and moderate downweight to all GP studies with high RoB


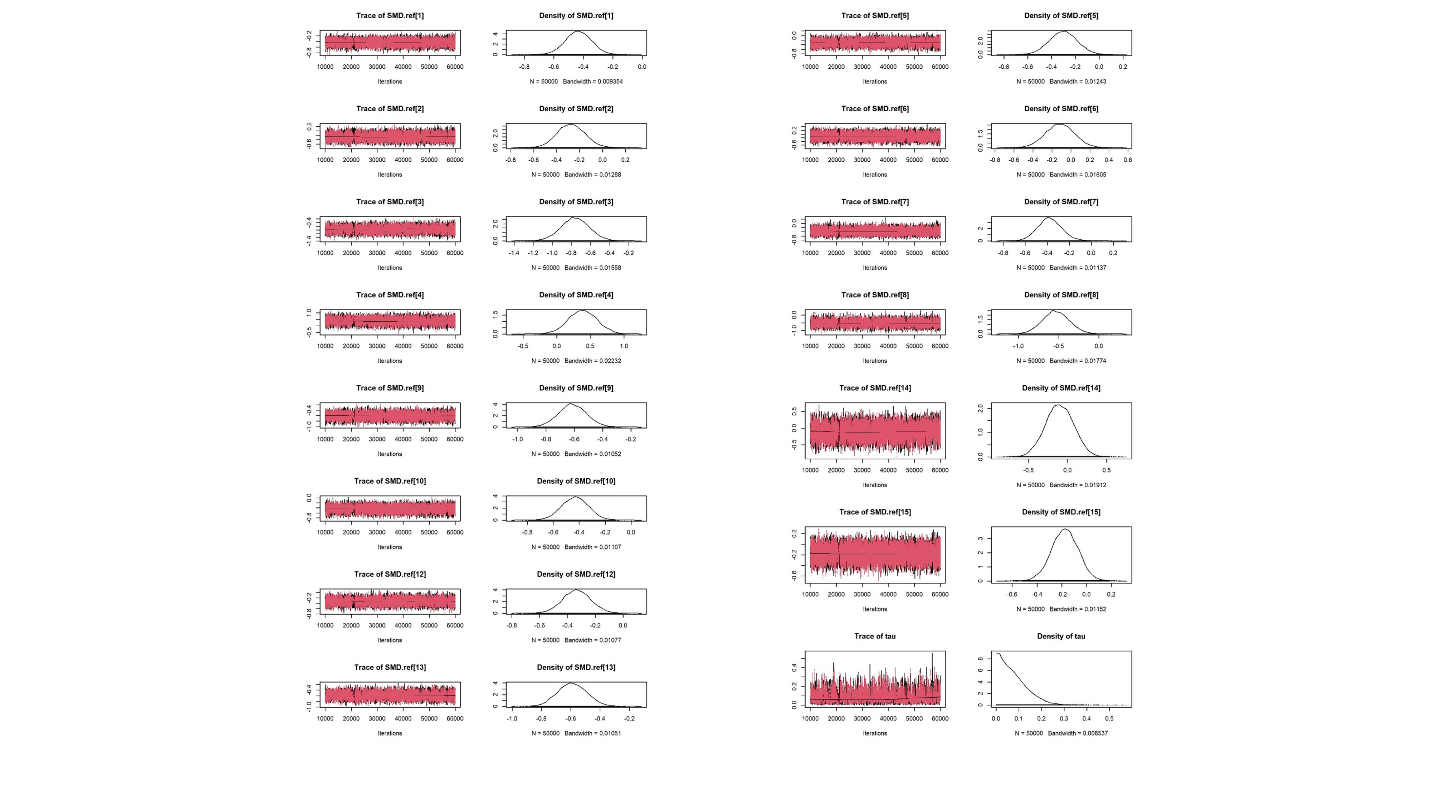


**Appendix Figure 13:** Trace plots for the NMA model with informative priors obtained from GP expert’s opinion for $\beta$ and moderate downweight to all GP studies with interventions in $T_{a}$-$T_{c}$

## League tables

**Appendix Table 10**: League table for all comparisons in CA network using a naive synthesis NMA model

|  | Aripiprazole | Asenapine | Clozapine | Fluphenazine | Haloperidol | Loxapine | Lurasidone | Molindone | Olanzapine | Paliperidone | Placebo | Quetiapine | Risperidone | Trifluoperazine | Ziprasidone |
| --- | --- | --- | --- | --- | --- | --- | --- | --- | --- | --- | --- | --- | --- | --- | --- |
| Aripiprazole | NA | -0.063(-0.176,0.05) | 0.456(0.286,0.616) | -0.466(-0.752,-0.181) | 0.017(-0.062,0.097) | -0.011(-0.19,0.165) | -0.086(-0.197,0.027) | 0.108(-0.163,0.382) | 0.153(0.074,0.23) | 0.055(-0.059,0.166) | -0.429(-0.501,-0.354) | -0.018(-0.112,0.075) | 0.102(0.018,0.188) | -0.108(-0.365,0.144) | -0.071(-0.17,0.027) |
| Asenapine | 0.063(-0.05,0.176) | NA | 0.519(0.348,0.703) | -0.403(-0.689,-0.12) | 0.08(-0.019,0.186) | 0.053(-0.136,0.253) | -0.022(-0.152,0.107) | 0.171(-0.12,0.456) | 0.216(0.116,0.315) | 0.119(-0.008,0.251) | -0.366(-0.46,-0.273) | 0.045(-0.073,0.165) | 0.165(0.06,0.27) | -0.044(-0.305,0.226) | -0.008(-0.132,0.113) |
| Clozapine | -0.456(-0.616,-0.286) | -0.519(-0.703,-0.348) | NA | -0.922(-1.23,-0.606) | -0.439(-0.593,-0.289) | -0.466(-0.689,-0.254) | -0.541(-0.722,-0.366) | -0.348(-0.645,-0.047) | -0.303(-0.46,-0.147) | -0.4(-0.574,-0.228) | -0.885(-1.039,-0.732) | -0.474(-0.631,-0.31) | -0.354(-0.509,-0.194) | -0.564(-0.839,-0.284) | -0.527(-0.688,-0.363) |
| Fluphenazine | 0.466(0.181,0.752) | 0.403(0.12,0.689) | 0.922(0.606,1.23) | NA | 0.483(0.208,0.758) | 0.455(0.142,0.762) | 0.381(0.078,0.664) | 0.574(0.204,0.963) | 0.619(0.335,0.894) | 0.521(0.241,0.821) | 0.037(-0.247,0.309) | 0.448(0.164,0.733) | 0.568(0.289,0.846) | 0.358(-0.009,0.725) | 0.395(0.108,0.677) |
| Haloperidol | -0.017(-0.097,0.062) | -0.08(-0.186,0.019) | 0.439(0.289,0.593) | -0.483(-0.758,-0.208) | NA | -0.027(-0.192,0.141) | -0.102(-0.198,-0.005) | 0.091(-0.182,0.358) | 0.136(0.073,0.196) | 0.039(-0.062,0.142) | -0.446(-0.505,-0.392) | -0.034(-0.119,0.043) | 0.086(0.021,0.146) | -0.124(-0.367,0.115) | -0.088(-0.177,-0.002) |
| Loxapine | 0.011(-0.165,0.19) | -0.053(-0.253,0.136) | 0.466(0.254,0.689) | -0.455(-0.762,-0.142) | 0.027(-0.141,0.192) | NA | -0.075(-0.271,0.115) | 0.118(-0.18,0.401) | 0.164(-0.014,0.337) | 0.066(-0.126,0.255) | -0.418(-0.584,-0.254) | -0.007(-0.185,0.171) | 0.113(-0.057,0.281) | -0.097(-0.326,0.132) | -0.06(-0.25,0.121) |
| Lurasidone | 0.086(-0.027,0.197) | 0.022(-0.107,0.152) | 0.541(0.366,0.722) | -0.381(-0.664,-0.078) | 0.102(0.005,0.198) | 0.075(-0.115,0.271) | NA | 0.193(-0.086,0.474) | 0.239(0.143,0.336) | 0.141(0.018,0.264) | -0.344(-0.43,-0.253) | 0.068(-0.044,0.176) | 0.188(0.093,0.284) | -0.022(-0.281,0.243) | 0.014(-0.099,0.136) |
| Molindone | -0.108(-0.382,0.163) | -0.171(-0.456,0.12) | 0.348(0.047,0.645) | -0.574(-0.963,-0.204) | -0.091(-0.358,0.182) | -0.118(-0.401,0.18) | -0.193(-0.474,0.086) | NA | 0.045(-0.218,0.314) | -0.053(-0.34,0.233) | -0.537(-0.8,-0.274) | -0.126(-0.399,0.152) | -0.006(-0.265,0.26) | -0.216(-0.533,0.104) | -0.179(-0.452,0.1) |
| Olanzapine | -0.153(-0.23,-0.074) | -0.216(-0.315,-0.116) | 0.303(0.147,0.46) | -0.619(-0.894,-0.335) | -0.136(-0.196,-0.073) | -0.164(-0.337,0.014) | -0.239(-0.336,-0.143) | -0.045(-0.314,0.218) | NA | -0.098(-0.192,-0.005) | -0.582(-0.634,-0.529) | -0.171(-0.25,-0.092) | -0.051(-0.114,0.012) | -0.261(-0.51,-0.021) | -0.224(-0.31,-0.137) |
| Paliperidone | -0.055(-0.166,0.059) | -0.119(-0.251,0.008) | 0.4(0.228,0.574) | -0.521(-0.821,-0.241) | -0.039(-0.142,0.062) | -0.066(-0.255,0.126) | -0.141(-0.264,-0.018) | 0.053(-0.233,0.34) | 0.098(0.005,0.192) | NA | -0.484(-0.58,-0.393) | -0.073(-0.186,0.035) | 0.047(-0.053,0.155) | -0.163(-0.432,0.084) | -0.126(-0.242,-0.005) |
| Placebo | 0.429(0.354,0.501) | 0.366(0.273,0.46) | 0.885(0.732,1.039) | -0.037(-0.309,0.247) | 0.446(0.392,0.505) | 0.418(0.254,0.584) | 0.344(0.253,0.43) | 0.537(0.274,0.8) | 0.582(0.529,0.634) | 0.484(0.393,0.58) | NA | 0.411(0.341,0.484) | 0.531(0.478,0.588) | 0.321(0.073,0.556) | 0.358(0.278,0.444) |
| Quetiapine | 0.018(-0.075,0.112) | -0.045(-0.165,0.073) | 0.474(0.31,0.631) | -0.448(-0.733,-0.164) | 0.034(-0.043,0.119) | 0.007(-0.171,0.185) | -0.068(-0.176,0.044) | 0.126(-0.152,0.399) | 0.171(0.092,0.25) | 0.073(-0.035,0.186) | -0.411(-0.484,-0.341) | NA | 0.12(0.039,0.197) | -0.09(-0.338,0.164) | -0.053(-0.154,0.051) |
| Risperidone | -0.102(-0.188,-0.018) | -0.165(-0.27,-0.06) | 0.354(0.194,0.509) | -0.568(-0.846,-0.289) | -0.086(-0.146,-0.021) | -0.113(-0.281,0.057) | -0.188(-0.284,-0.093) | 0.006(-0.26,0.265) | 0.051(-0.012,0.114) | -0.047(-0.155,0.053) | -0.531(-0.588,-0.478) | -0.12(-0.197,-0.039) | NA | -0.21(-0.455,0.035) | -0.173(-0.263,-0.082) |
| Trifluoperazine | 0.108(-0.144,0.365) | 0.044(-0.226,0.305) | 0.564(0.284,0.839) | -0.358(-0.725,0.009) | 0.124(-0.115,0.367) | 0.097(-0.132,0.326) | 0.022(-0.243,0.281) | 0.216(-0.104,0.533) | 0.261(0.021,0.51) | 0.163(-0.084,0.432) | -0.321(-0.556,-0.073) | 0.09(-0.164,0.338) | 0.21(-0.035,0.455) | NA | 0.037(-0.214,0.295) |
| Ziprasidone | 0.071(-0.027,0.17) | 0.008(-0.113,0.132) | 0.527(0.363,0.688) | -0.395(-0.677,-0.108) | 0.088(0.002,0.177) | 0.06(-0.121,0.25) | -0.014(-0.136,0.099) | 0.179(-0.1,0.452) | 0.224(0.137,0.31) | 0.126(0.005,0.242) | -0.358(-0.444,-0.278) | 0.053(-0.051,0.154) | 0.173(0.082,0.263) | -0.037(-0.295,0.214) | NA |

|  | Aripiprazole | Asenapine | Clozapine | Fluphenazine | Haloperidol | Loxapine | Lurasidone | Molindone | Olanzapine | Paliperidone | Placebo | Quetiapine | Risperidone | Trifluoperazine | Ziprasidone |
| --- | --- | --- | --- | --- | --- | --- | --- | --- | --- | --- | --- | --- | --- | --- | --- |
| Aripiprazole | NA | -0.066(-0.33,0.199) | 0.455(0.122,0.782) | -0.54(-0.96,-0.137) | 0.039(-0.233,0.304) | -0.034(-0.393,0.313) | -0.038(-0.295,0.206) | 0.178(-0.207,0.541) | 0.198(-0.056,0.449) | -0.012(-0.221,0.192) | -0.429(-0.599,-0.264) | -0.036(-0.27,0.195) | 0.121(-0.151,0.383) | 0.005(-0.523,0.557) | -0.151(-0.452,0.17) |
| Asenapine | 0.066(-0.199,0.33) | NA | 0.521(0.171,0.876) | -0.474(-0.911,-0.051) | 0.105(-0.19,0.382) | 0.032(-0.338,0.416) | 0.028(-0.257,0.305) | 0.245(-0.145,0.625) | 0.264(-0.025,0.526) | 0.054(-0.219,0.34) | -0.363(-0.576,-0.157) | 0.03(-0.253,0.311) | 0.187(-0.117,0.471) | 0.071(-0.492,0.625) | -0.085(-0.43,0.258) |
| Clozapine | -0.455(-0.782,-0.122) | -0.521(-0.876,-0.171) | NA | -0.995(-1.498,-0.507) | -0.416(-0.762,-0.058) | -0.489(-0.906,-0.076) | -0.493(-0.838,-0.145) | -0.277(-0.7,0.127) | -0.257(-0.58,0.08) | -0.467(-0.813,-0.119) | -0.884(-1.169,-0.606) | -0.491(-0.825,-0.154) | -0.334(-0.685,0.035) | -0.45(-1.045,0.14) | -0.606(-0.983,-0.196) |
| Fluphenazine | 0.54(0.137,0.96) | 0.474(0.051,0.911) | 0.995(0.507,1.498) | NA | 0.579(0.169,0.991) | 0.506(0.018,1.006) | 0.502(0.099,0.923) | 0.719(0.227,1.21) | 0.738(0.32,1.166) | 0.528(0.12,0.953) | 0.111(-0.246,0.489) | 0.504(0.106,0.948) | 0.661(0.227,1.089) | 0.545(-0.089,1.177) | 0.389(-0.05,0.845) |
| Haloperidol | -0.039(-0.304,0.233) | -0.105(-0.382,0.19) | 0.416(0.058,0.762) | -0.579(-0.991,-0.169) | NA | -0.073(-0.402,0.273) | -0.077(-0.362,0.196) | 0.139(-0.244,0.491) | 0.159(-0.105,0.411) | -0.051(-0.338,0.237) | -0.468(-0.676,-0.27) | -0.075(-0.348,0.208) | 0.082(-0.213,0.361) | -0.034(-0.578,0.51) | -0.19(-0.519,0.131) |
| Loxapine | 0.034(-0.313,0.393) | -0.032(-0.416,0.338) | 0.489(0.076,0.906) | -0.506(-1.006,-0.018) | 0.073(-0.273,0.402) | NA | -0.004(-0.372,0.366) | 0.212(-0.253,0.654) | 0.232(-0.13,0.592) | 0.022(-0.318,0.381) | -0.395(-0.705,-0.09) | -0.002(-0.341,0.354) | 0.155(-0.222,0.529) | 0.039(-0.386,0.469) | -0.117(-0.511,0.309) |
| Lurasidone | 0.038(-0.206,0.295) | -0.028(-0.305,0.257) | 0.493(0.145,0.838) | -0.502(-0.923,-0.099) | 0.077(-0.196,0.362) | 0.004(-0.366,0.372) | NA | 0.217(-0.159,0.597) | 0.237(-0.029,0.493) | 0.026(-0.259,0.305) | -0.391(-0.576,-0.199) | 0.003(-0.26,0.27) | 0.16(-0.129,0.447) | 0.043(-0.497,0.577) | -0.113(-0.406,0.229) |
| Molindone | -0.178(-0.541,0.207) | -0.245(-0.625,0.145) | 0.277(-0.127,0.7) | -0.719(-1.21,-0.227) | -0.139(-0.491,0.244) | -0.212(-0.654,0.253) | -0.217(-0.597,0.159) | NA | 0.02(-0.298,0.35) | -0.19(-0.57,0.182) | -0.607(-0.925,-0.277) | -0.214(-0.575,0.161) | -0.057(-0.401,0.296) | -0.173(-0.769,0.417) | -0.329(-0.707,0.117) |
| Olanzapine | -0.198(-0.449,0.056) | -0.264(-0.526,0.025) | 0.257(-0.08,0.58) | -0.738(-1.166,-0.32) | -0.159(-0.411,0.105) | -0.232(-0.592,0.13) | -0.237(-0.493,0.029) | -0.02(-0.35,0.298) | NA | -0.21(-0.488,0.055) | -0.627(-0.81,-0.429) | -0.234(-0.493,0.028) | -0.077(-0.326,0.163) | -0.193(-0.748,0.342) | -0.349(-0.647,-0.017) |
| Paliperidone | 0.012(-0.192,0.221) | -0.054(-0.34,0.219) | 0.467(0.119,0.813) | -0.528(-0.953,-0.12) | 0.051(-0.237,0.338) | -0.022(-0.381,0.318) | -0.026(-0.305,0.259) | 0.19(-0.182,0.57) | 0.21(-0.055,0.488) | NA | -0.417(-0.617,-0.215) | -0.024(-0.28,0.252) | 0.133(-0.151,0.415) | 0.017(-0.539,0.56) | -0.139(-0.454,0.193) |
| Placebo | 0.429(0.264,0.599) | 0.363(0.157,0.576) | 0.884(0.606,1.169) | -0.111(-0.489,0.246) | 0.468(0.27,0.676) | 0.395(0.09,0.705) | 0.391(0.199,0.576) | 0.607(0.277,0.925) | 0.627(0.429,0.81) | 0.417(0.215,0.617) | NA | 0.393(0.206,0.571) | 0.55(0.333,0.748) | 0.434(-0.096,0.954) | 0.278(0.025,0.546) |
| Quetiapine | 0.036(-0.195,0.27) | -0.03(-0.311,0.253) | 0.491(0.154,0.825) | -0.504(-0.948,-0.106) | 0.075(-0.208,0.348) | 0.002(-0.354,0.341) | -0.003(-0.27,0.26) | 0.214(-0.161,0.575) | 0.234(-0.028,0.493) | 0.024(-0.252,0.28) | -0.393(-0.571,-0.206) | NA | 0.157(-0.109,0.429) | 0.041(-0.5,0.573) | -0.115(-0.409,0.219) |
| Risperidone | -0.121(-0.383,0.151) | -0.187(-0.471,0.117) | 0.334(-0.035,0.685) | -0.661(-1.089,-0.227) | -0.082(-0.361,0.213) | -0.155(-0.529,0.222) | -0.16(-0.447,0.129) | 0.057(-0.296,0.401) | 0.077(-0.163,0.326) | -0.133(-0.415,0.151) | -0.55(-0.748,-0.333) | -0.157(-0.429,0.109) | NA | -0.116(-0.661,0.439) | -0.272(-0.578,0.087) |
| Trifluoperazine | -0.005(-0.557,0.523) | -0.071(-0.625,0.492) | 0.45(-0.14,1.045) | -0.545(-1.177,0.089) | 0.034(-0.51,0.578) | -0.039(-0.469,0.386) | -0.043(-0.577,0.497) | 0.173(-0.417,0.769) | 0.193(-0.342,0.748) | -0.017(-0.56,0.539) | -0.434(-0.954,0.096) | -0.041(-0.573,0.5) | 0.116(-0.439,0.661) | NA | -0.156(-0.729,0.43) |
| Ziprasidone | 0.151(-0.17,0.452) | 0.085(-0.258,0.43) | 0.606(0.196,0.983) | -0.389(-0.845,0.05) | 0.19(-0.131,0.519) | 0.117(-0.309,0.511) | 0.113(-0.229,0.406) | 0.329(-0.117,0.707) | 0.349(0.017,0.647) | 0.139(-0.193,0.454) | -0.278(-0.546,-0.025) | 0.115(-0.219,0.409) | 0.272(-0.087,0.578) | 0.156(-0.43,0.729) | NA |

**Appendix Table 11**: League table for all comparisons in CA network. Informative priors from GP studies using a data based approach for β and no downweight for GP studies.

|  | Aripiprazole | Asenapine | Clozapine | Fluphenazine | Haloperidol | Loxapine | Lurasidone | Molindone | Olanzapine | Paliperidone | Placebo | Quetiapine | Risperidone | Trifluoperazine | Ziprasidone |
| --- | --- | --- | --- | --- | --- | --- | --- | --- | --- | --- | --- | --- | --- | --- | --- |
| Aripiprazole | NA | -0.071(-0.338,0.185) | 0.465(0.127,0.813) | -0.523(-0.918,-0.133) | 0.045(-0.226,0.3) | -0.026(-0.374,0.327) | -0.033(-0.289,0.219) | 0.176(-0.201,0.545) | 0.205(-0.045,0.458) | -0.012(-0.233,0.209) | -0.429(-0.592,-0.254) | -0.04(-0.272,0.193) | 0.124(-0.146,0.376) | 0.017(-0.498,0.538) | -0.143(-0.451,0.174) |
| Asenapine | 0.071(-0.185,0.338) | NA | 0.536(0.185,0.906) | -0.452(-0.867,-0.032) | 0.116(-0.163,0.395) | 0.045(-0.325,0.421) | 0.038(-0.248,0.318) | 0.247(-0.145,0.642) | 0.275(-0.009,0.548) | 0.059(-0.219,0.344) | -0.358(-0.563,-0.138) | 0.03(-0.234,0.327) | 0.195(-0.092,0.496) | 0.088(-0.436,0.662) | -0.072(-0.368,0.278) |
| Clozapine | -0.465(-0.813,-0.127) | -0.536(-0.906,-0.185) | NA | -0.988(-1.459,-0.52) | -0.42(-0.745,-0.086) | -0.491(-0.906,-0.047) | -0.498(-0.862,-0.137) | -0.289(-0.737,0.132) | -0.26(-0.601,0.072) | -0.477(-0.844,-0.12) | -0.894(-1.202,-0.596) | -0.505(-0.877,-0.147) | -0.341(-0.704,0.025) | -0.448(-1.013,0.141) | -0.608(-1.007,-0.21) |
| Fluphenazine | 0.523(0.133,0.918) | 0.452(0.032,0.867) | 0.988(0.52,1.459) | NA | 0.568(0.179,0.96) | 0.497(0.026,0.972) | 0.49(0.074,0.914) | 0.699(0.213,1.193) | 0.728(0.318,1.156) | 0.511(0.083,0.922) | 0.094(-0.275,0.465) | 0.483(0.076,0.897) | 0.647(0.224,1.054) | 0.54(-0.063,1.135) | 0.38(-0.063,0.844) |
| Haloperidol | -0.045(-0.3,0.226) | -0.116(-0.395,0.163) | 0.42(0.086,0.745) | -0.568(-0.96,-0.179) | NA | -0.071(-0.421,0.263) | -0.078(-0.379,0.208) | 0.131(-0.261,0.506) | 0.16(-0.092,0.421) | -0.057(-0.343,0.226) | -0.474(-0.675,-0.264) | -0.085(-0.373,0.199) | 0.079(-0.214,0.355) | -0.028(-0.553,0.485) | -0.188(-0.509,0.166) |
| Loxapine | 0.026(-0.327,0.374) | -0.045(-0.421,0.325) | 0.491(0.047,0.906) | -0.497(-0.972,-0.026) | 0.071(-0.263,0.421) | NA | -0.007(-0.387,0.36) | 0.202(-0.26,0.651) | 0.231(-0.124,0.571) | 0.014(-0.361,0.391) | -0.403(-0.71,-0.094) | -0.014(-0.389,0.338) | 0.15(-0.219,0.516) | 0.043(-0.373,0.433) | -0.117(-0.532,0.294) |
| Lurasidone | 0.033(-0.219,0.289) | -0.038(-0.318,0.248) | 0.498(0.137,0.862) | -0.49(-0.914,-0.074) | 0.078(-0.208,0.379) | 0.007(-0.36,0.387) | NA | 0.208(-0.171,0.591) | 0.237(-0.021,0.494) | 0.021(-0.262,0.321) | -0.396(-0.588,-0.202) | -0.008(-0.278,0.268) | 0.157(-0.115,0.428) | 0.05(-0.478,0.598) | -0.11(-0.411,0.242) |
| Molindone | -0.176(-0.545,0.201) | -0.247(-0.642,0.145) | 0.289(-0.132,0.737) | -0.699(-1.193,-0.213) | -0.131(-0.506,0.261) | -0.202(-0.651,0.26) | -0.208(-0.591,0.171) | NA | 0.029(-0.3,0.374) | -0.188(-0.565,0.202) | -0.605(-0.935,-0.262) | -0.216(-0.586,0.186) | -0.052(-0.41,0.286) | -0.159(-0.765,0.444) | -0.319(-0.736,0.127) |
| Olanzapine | -0.205(-0.458,0.045) | -0.275(-0.548,0.009) | 0.26(-0.072,0.601) | -0.728(-1.156,-0.318) | -0.16(-0.421,0.092) | -0.231(-0.571,0.124) | -0.237(-0.494,0.021) | -0.029(-0.374,0.3) | NA | -0.216(-0.471,0.056) | -0.634(-0.817,-0.454) | -0.245(-0.504,0.016) | -0.081(-0.327,0.168) | -0.188(-0.721,0.347) | -0.348(-0.649,-0.02) |
| Paliperidone | 0.012(-0.209,0.233) | -0.059(-0.344,0.219) | 0.477(0.12,0.844) | -0.511(-0.922,-0.083) | 0.057(-0.226,0.343) | -0.014(-0.391,0.361) | -0.021(-0.321,0.262) | 0.188(-0.202,0.565) | 0.216(-0.056,0.471) | NA | -0.417(-0.618,-0.218) | -0.028(-0.302,0.233) | 0.136(-0.165,0.41) | 0.029(-0.513,0.561) | -0.131(-0.45,0.193) |
| Placebo | 0.429(0.254,0.592) | 0.358(0.138,0.563) | 0.894(0.596,1.202) | -0.094(-0.465,0.275) | 0.474(0.264,0.675) | 0.403(0.094,0.71) | 0.396(0.202,0.588) | 0.605(0.262,0.935) | 0.634(0.454,0.817) | 0.417(0.218,0.618) | NA | 0.389(0.2,0.576) | 0.553(0.336,0.752) | 0.446(-0.054,0.957) | 0.286(0.042,0.558) |
| Quetiapine | 0.04(-0.193,0.272) | -0.03(-0.327,0.234) | 0.505(0.147,0.877) | -0.483(-0.897,-0.076) | 0.085(-0.199,0.373) | 0.014(-0.338,0.389) | 0.008(-0.268,0.278) | 0.216(-0.186,0.586) | 0.245(-0.016,0.504) | 0.028(-0.233,0.302) | -0.389(-0.576,-0.2) | NA | 0.164(-0.106,0.426) | 0.057(-0.486,0.602) | -0.103(-0.422,0.222) |
| Risperidone | -0.124(-0.376,0.146) | -0.195(-0.496,0.092) | 0.341(-0.025,0.704) | -0.647(-1.054,-0.224) | -0.079(-0.355,0.214) | -0.15(-0.516,0.219) | -0.157(-0.428,0.115) | 0.052(-0.286,0.41) | 0.081(-0.168,0.327) | -0.136(-0.41,0.165) | -0.553(-0.752,-0.336) | -0.164(-0.426,0.106) | NA | -0.107(-0.636,0.446) | -0.267(-0.587,0.095) |
| Trifluoperazine | -0.017(-0.538,0.498) | -0.088(-0.662,0.436) | 0.448(-0.141,1.013) | -0.54(-1.135,0.063) | 0.028(-0.485,0.553) | -0.043(-0.433,0.373) | -0.05(-0.598,0.478) | 0.159(-0.444,0.765) | 0.188(-0.347,0.721) | -0.029(-0.561,0.513) | -0.446(-0.957,0.054) | -0.057(-0.602,0.486) | 0.107(-0.446,0.636) | NA | -0.16(-0.729,0.397) |
| Ziprasidone | 0.143(-0.174,0.451) | 0.072(-0.278,0.368) | 0.608(0.21,1.007) | -0.38(-0.844,0.063) | 0.188(-0.166,0.509) | 0.117(-0.294,0.532) | 0.11(-0.242,0.411) | 0.319(-0.127,0.736) | 0.348(0.02,0.649) | 0.131(-0.193,0.45) | -0.286(-0.558,-0.042) | 0.103(-0.222,0.422) | 0.267(-0.095,0.587) | 0.16(-0.397,0.729) | NA |

**Appendix Table 12:** League table for all comparisons in CA network. Informative priors from GP studies using a data based approach for $\beta$ and moderate downweight for GP studies with high RoB.

|  | Aripiprazole | Asenapine | Clozapine | Fluphenazine | Haloperidol | Loxapine | Lurasidone | Molindone | Olanzapine | Paliperidone | Placebo | Quetiapine | Risperidone | Trifluoperazine | Ziprasidone |
| --- | --- | --- | --- | --- | --- | --- | --- | --- | --- | --- | --- | --- | --- | --- | --- |
| Aripiprazole | NA | -0.065(-0.311,0.18) | 0.45(0.167,0.752) | -0.563(-0.959,-0.172) | 0.045(-0.184,0.282) | -0.021(-0.362,0.309) | -0.044(-0.269,0.185) | 0.159(-0.213,0.526) | 0.191(-0.039,0.423) | -0.009(-0.203,0.194) | -0.43(-0.578,-0.272) | -0.028(-0.242,0.191) | 0.101(-0.145,0.36) | 0.017(-0.468,0.557) | -0.146(-0.435,0.205) |
| Asenapine | 0.065(-0.18,0.311) | NA | 0.515(0.2,0.828) | -0.498(-0.898,-0.11) | 0.11(-0.137,0.363) | 0.044(-0.325,0.386) | 0.021(-0.229,0.265) | 0.224(-0.152,0.596) | 0.256(0.002,0.511) | 0.056(-0.215,0.306) | -0.365(-0.555,-0.174) | 0.037(-0.227,0.29) | 0.166(-0.11,0.437) | 0.082(-0.442,0.609) | -0.082(-0.395,0.266) |
| Clozapine | -0.45(-0.752,-0.167) | -0.515(-0.828,-0.2) | NA | -1.013(-1.46,-0.578) | -0.405(-0.701,-0.127) | -0.471(-0.847,-0.086) | -0.494(-0.808,-0.197) | -0.291(-0.705,0.115) | -0.259(-0.547,0.036) | -0.459(-0.784,-0.15) | -0.88(-1.132,-0.626) | -0.478(-0.795,-0.171) | -0.349(-0.667,-0.041) | -0.433(-0.976,0.132) | -0.597(-0.942,-0.218) |
| Fluphenazine | 0.563(0.172,0.959) | 0.498(0.11,0.898) | 1.013(0.578,1.46) | NA | 0.608(0.236,1.005) | 0.542(0.084,1.019) | 0.519(0.127,0.929) | 0.722(0.234,1.184) | 0.754(0.362,1.146) | 0.555(0.15,0.943) | 0.133(-0.208,0.499) | 0.535(0.123,0.936) | 0.664(0.252,1.064) | 0.581(-0.016,1.192) | 0.417(-0.002,0.867) |
| Haloperidol | -0.045(-0.282,0.184) | -0.11(-0.363,0.137) | 0.405(0.127,0.701) | -0.608(-1.005,-0.236) | NA | -0.066(-0.388,0.246) | -0.089(-0.336,0.152) | 0.114(-0.273,0.483) | 0.146(-0.082,0.378) | -0.053(-0.295,0.198) | -0.475(-0.64,-0.299) | -0.073(-0.327,0.173) | 0.056(-0.21,0.319) | -0.028(-0.532,0.49) | -0.191(-0.504,0.144) |
| Loxapine | 0.021(-0.309,0.362) | -0.044(-0.386,0.325) | 0.471(0.086,0.847) | -0.542(-1.019,-0.084) | 0.066(-0.246,0.388) | NA | -0.023(-0.362,0.294) | 0.18(-0.273,0.629) | 0.212(-0.114,0.549) | 0.012(-0.336,0.358) | -0.409(-0.697,-0.106) | -0.007(-0.349,0.354) | 0.122(-0.229,0.477) | 0.038(-0.337,0.444) | -0.126(-0.508,0.298) |
| Lurasidone | 0.044(-0.185,0.269) | -0.021(-0.265,0.229) | 0.494(0.197,0.808) | -0.519(-0.929,-0.127) | 0.089(-0.152,0.336) | 0.023(-0.294,0.362) | NA | 0.203(-0.167,0.566) | 0.235(0.003,0.475) | 0.035(-0.224,0.294) | -0.386(-0.554,-0.202) | 0.016(-0.246,0.26) | 0.145(-0.121,0.409) | 0.061(-0.453,0.594) | -0.103(-0.395,0.246) |
| Molindone | -0.159(-0.526,0.213) | -0.224(-0.596,0.152) | 0.291(-0.115,0.705) | -0.722(-1.184,-0.234) | -0.114(-0.483,0.273) | -0.18(-0.629,0.273) | -0.203(-0.566,0.167) | NA | 0.032(-0.287,0.36) | -0.168(-0.545,0.223) | -0.589(-0.915,-0.257) | -0.187(-0.555,0.188) | -0.058(-0.407,0.276) | -0.142(-0.758,0.473) | -0.306(-0.717,0.142) |
| Olanzapine | -0.191(-0.423,0.039) | -0.256(-0.511,-0.002) | 0.259(-0.036,0.547) | -0.754(-1.146,-0.362) | -0.146(-0.378,0.082) | -0.212(-0.549,0.114) | -0.235(-0.475,-0.003) | -0.032(-0.36,0.287) | NA | -0.2(-0.436,0.051) | -0.621(-0.788,-0.459) | -0.219(-0.47,0.028) | -0.09(-0.327,0.133) | -0.174(-0.668,0.35) | -0.338(-0.63,0.011) |
| Paliperidone | 0.009(-0.194,0.203) | -0.056(-0.306,0.215) | 0.459(0.15,0.784) | -0.555(-0.943,-0.15) | 0.053(-0.198,0.295) | -0.012(-0.358,0.336) | -0.035(-0.294,0.224) | 0.168(-0.223,0.545) | 0.2(-0.051,0.436) | NA | -0.421(-0.602,-0.24) | -0.02(-0.275,0.232) | 0.109(-0.165,0.377) | 0.026(-0.479,0.549) | -0.138(-0.436,0.195) |
| Placebo | 0.43(0.272,0.578) | 0.365(0.174,0.555) | 0.88(0.626,1.132) | -0.133(-0.499,0.208) | 0.475(0.299,0.64) | 0.409(0.106,0.697) | 0.386(0.202,0.554) | 0.589(0.257,0.915) | 0.621(0.459,0.788) | 0.421(0.24,0.602) | NA | 0.402(0.221,0.576) | 0.531(0.33,0.725) | 0.447(-0.013,0.936) | 0.283(0.037,0.567) |
| Quetiapine | 0.028(-0.191,0.242) | -0.037(-0.29,0.227) | 0.478(0.171,0.795) | -0.535(-0.936,-0.123) | 0.073(-0.173,0.327) | 0.007(-0.354,0.349) | -0.016(-0.26,0.246) | 0.187(-0.188,0.555) | 0.219(-0.028,0.47) | 0.02(-0.232,0.275) | -0.402(-0.576,-0.221) | NA | 0.129(-0.129,0.394) | 0.045(-0.459,0.589) | -0.118(-0.424,0.215) |
| Risperidone | -0.101(-0.36,0.145) | -0.166(-0.437,0.11) | 0.349(0.041,0.667) | -0.664(-1.064,-0.252) | -0.056(-0.319,0.21) | -0.122(-0.477,0.229) | -0.145(-0.409,0.121) | 0.058(-0.276,0.407) | 0.09(-0.133,0.327) | -0.109(-0.377,0.165) | -0.531(-0.725,-0.33) | -0.129(-0.394,0.129) | NA | -0.084(-0.583,0.451) | -0.247(-0.551,0.121) |
| Trifluoperazine | -0.017(-0.557,0.468) | -0.082(-0.609,0.442) | 0.433(-0.132,0.976) | -0.581(-1.192,0.016) | 0.028(-0.49,0.532) | -0.038(-0.444,0.337) | -0.061(-0.594,0.453) | 0.142(-0.473,0.758) | 0.174(-0.35,0.668) | -0.026(-0.549,0.479) | -0.447(-0.936,0.013) | -0.045(-0.589,0.459) | 0.084(-0.451,0.583) | NA | -0.164(-0.703,0.378) |
| Ziprasidone | 0.146(-0.205,0.435) | 0.082(-0.266,0.395) | 0.597(0.218,0.942) | -0.417(-0.867,0.002) | 0.191(-0.144,0.504) | 0.126(-0.298,0.508) | 0.103(-0.246,0.395) | 0.306(-0.142,0.717) | 0.338(-0.011,0.63) | 0.138(-0.195,0.436) | -0.283(-0.567,-0.037) | 0.118(-0.215,0.424) | 0.247(-0.121,0.551) | 0.164(-0.378,0.703) | NA |

**Appendix Table 13:** League table for all comparisons in CA network. Informative priors from GP studies using a data based approach for $\beta$ and moderate downweight for GP studies evaluating interventions in $T_{\alpha}-T_{c}$.

|  | Aripiprazole | Asenapine | Clozapine | Fluphenazine | Haloperidol | Loxapine | Lurasidone | Molindone | Olanzapine | Paliperidone | Placebo | Quetiapine | Risperidone | Trifluoperazine | Ziprasidone |
| --- | --- | --- | --- | --- | --- | --- | --- | --- | --- | --- | --- | --- | --- | --- | --- |
| Aripiprazole | NA | -0.154(-0.471,0.149) | 0.347(-0.021,0.689) | -0.791(-1.238,-0.338) | -0.122(-0.435,0.181) | -0.378(-0.736,-0.011) | -0.046(-0.341,0.227) | 0.137(-0.257,0.54) | 0.189(-0.088,0.471) | -0.01(-0.234,0.203) | -0.434(-0.62,-0.256) | -0.095(-0.344,0.151) | 0.16(-0.119,0.421) | -0.369(-0.792,0.032) | -0.26(-0.546,0.025) |
| Asenapine | 0.154(-0.149,0.471) | NA | 0.501(0.106,0.904) | -0.636(-1.115,-0.125) | 0.032(-0.308,0.39) | -0.223(-0.615,0.183) | 0.109(-0.214,0.432) | 0.291(-0.117,0.721) | 0.344(0.025,0.669) | 0.145(-0.17,0.484) | -0.28(-0.516,-0.02) | 0.059(-0.236,0.366) | 0.315(0.018,0.649) | -0.214(-0.645,0.227) | -0.106(-0.435,0.226) |
| Clozapine | -0.347(-0.689,0.021) | -0.501(-0.904,-0.106) | NA | -1.138(-1.645,-0.601) | -0.469(-0.84,-0.074) | -0.725(-1.14,-0.284) | -0.393(-0.777,-0.028) | -0.21(-0.661,0.287) | -0.157(-0.522,0.22) | -0.357(-0.726,0.016) | -0.781(-1.08,-0.46) | -0.442(-0.819,-0.069) | -0.187(-0.559,0.171) | -0.716(-1.176,-0.206) | -0.607(-0.993,-0.232) |
| Fluphenazine | 0.791(0.338,1.238) | 0.636(0.125,1.115) | 1.138(0.601,1.645) | NA | 0.668(0.22,1.117) | 0.413(-0.102,0.902) | 0.745(0.259,1.198) | 0.928(0.373,1.475) | 0.98(0.514,1.435) | 0.781(0.312,1.221) | 0.356(-0.082,0.755) | 0.695(0.226,1.153) | 0.951(0.486,1.4) | 0.422(-0.153,0.964) | 0.53(0.023,0.976) |
| Haloperidol | 0.122(-0.181,0.435) | -0.032(-0.39,0.308) | 0.469(0.074,0.84) | -0.668(-1.117,-0.22) | NA | -0.255(-0.61,0.095) | 0.077(-0.255,0.395) | 0.259(-0.151,0.67) | 0.312(0.017,0.62) | 0.113(-0.224,0.438) | -0.312(-0.56,-0.069) | 0.027(-0.295,0.331) | 0.283(-0.025,0.578) | -0.246(-0.669,0.166) | -0.138(-0.471,0.192) |
| Loxapine | 0.378(0.011,0.736) | 0.223(-0.183,0.615) | 0.725(0.284,1.14) | -0.413(-0.902,0.102) | 0.255(-0.095,0.61) | NA | 0.332(-0.047,0.736) | 0.515(0.035,0.993) | 0.567(0.18,0.953) | 0.368(-0.014,0.771) | -0.057(-0.357,0.271) | 0.282(-0.089,0.645) | 0.538(0.156,0.903) | 0.009(-0.313,0.343) | 0.117(-0.273,0.519) |
| Lurasidone | 0.046(-0.227,0.341) | -0.109(-0.432,0.214) | 0.393(0.028,0.777) | -0.745(-1.198,-0.259) | -0.077(-0.395,0.255) | -0.332(-0.736,0.047) | NA | 0.183(-0.232,0.594) | 0.235(-0.062,0.542) | 0.036(-0.27,0.353) | -0.388(-0.6,-0.163) | -0.05(-0.349,0.255) | 0.206(-0.074,0.497) | -0.323(-0.761,0.095) | -0.215(-0.524,0.1) |
| Molindone | -0.137(-0.54,0.257) | -0.291(-0.721,0.117) | 0.21(-0.287,0.661) | -0.928(-1.475,-0.373) | -0.259(-0.67,0.151) | -0.515(-0.993,-0.035) | -0.183(-0.594,0.232) | NA | 0.052(-0.318,0.413) | -0.147(-0.541,0.273) | -0.571(-0.941,-0.213) | -0.232(-0.644,0.165) | 0.023(-0.353,0.378) | -0.506(-1.008,0.016) | -0.397(-0.831,0.044) |
| Olanzapine | -0.189(-0.471,0.088) | -0.344(-0.669,-0.025) | 0.157(-0.22,0.522) | -0.98(-1.435,-0.514) | -0.312(-0.62,-0.017) | -0.567(-0.953,-0.18) | -0.235(-0.542,0.062) | -0.052(-0.413,0.318) | NA | -0.199(-0.49,0.09) | -0.624(-0.833,-0.415) | -0.285(-0.576,-0.011) | -0.029(-0.286,0.22) | -0.558(-0.974,-0.126) | -0.45(-0.762,-0.14) |
| Paliperidone | 0.01(-0.203,0.234) | -0.145(-0.484,0.17) | 0.357(-0.016,0.726) | -0.781(-1.221,-0.312) | -0.113(-0.438,0.224) | -0.368(-0.771,0.014) | -0.036(-0.353,0.27) | 0.147(-0.273,0.541) | 0.199(-0.09,0.49) | NA | -0.425(-0.643,-0.213) | -0.086(-0.375,0.181) | 0.17(-0.134,0.453) | -0.359(-0.781,0.088) | -0.251(-0.557,0.062) |
| Placebo | 0.434(0.256,0.62) | 0.28(0.02,0.516) | 0.781(0.46,1.08) | -0.356(-0.755,0.082) | 0.312(0.069,0.56) | 0.057(-0.271,0.357) | 0.388(0.163,0.6) | 0.571(0.213,0.941) | 0.624(0.415,0.833) | 0.425(0.213,0.643) | NA | 0.339(0.129,0.545) | 0.595(0.399,0.791) | 0.066(-0.316,0.435) | 0.174(-0.044,0.402) |
| Quetiapine | 0.095(-0.151,0.344) | -0.059(-0.366,0.236) | 0.442(0.069,0.819) | -0.695(-1.153,-0.226) | -0.027(-0.331,0.295) | -0.282(-0.645,0.089) | 0.05(-0.255,0.349) | 0.232(-0.165,0.644) | 0.285(0.011,0.576) | 0.086(-0.181,0.375) | -0.339(-0.545,-0.129) | NA | 0.256(-0.006,0.532) | -0.273(-0.714,0.162) | -0.165(-0.447,0.149) |
| Risperidone | -0.16(-0.421,0.119) | -0.315(-0.649,-0.018) | 0.187(-0.171,0.559) | -0.951(-1.4,-0.486) | -0.283(-0.578,0.025) | -0.538(-0.903,-0.156) | -0.206(-0.497,0.074) | -0.023(-0.378,0.353) | 0.029(-0.22,0.286) | -0.17(-0.453,0.134) | -0.595(-0.791,-0.399) | -0.256(-0.532,0.006) | NA | -0.529(-0.954,-0.108) | -0.421(-0.72,-0.129) |
| Trifluoperazine | 0.369(-0.032,0.792) | 0.214(-0.227,0.645) | 0.716(0.206,1.176) | -0.422(-0.964,0.153) | 0.246(-0.166,0.669) | -0.009(-0.343,0.313) | 0.323(-0.095,0.761) | 0.506(-0.016,1.008) | 0.558(0.126,0.974) | 0.359(-0.088,0.781) | -0.066(-0.435,0.316) | 0.273(-0.162,0.714) | 0.529(0.108,0.954) | NA | 0.108(-0.324,0.539) |
| Ziprasidone | 0.26(-0.025,0.546) | 0.106(-0.226,0.435) | 0.607(0.232,0.993) | -0.53(-0.976,-0.023) | 0.138(-0.192,0.471) | -0.117(-0.519,0.273) | 0.215(-0.1,0.524) | 0.397(-0.044,0.831) | 0.45(0.14,0.762) | 0.251(-0.062,0.557) | -0.174(-0.402,0.044) | 0.165(-0.149,0.447) | 0.421(0.129,0.72) | -0.108(-0.539,0.324) | NA |

**Appendix Table 14:** League table for all comparisons in CA network. Informative priors from GP studies using expert’s opinion for β and no downweight for GP studies.

|  | Aripiprazole | Asenapine | Clozapine | Fluphenazine | Haloperidol | Loxapine | Lurasidone | Molindone | Olanzapine | Paliperidone | Placebo | Quetiapine | Risperidone | Trifluoperazine | Ziprasidone |
| --- | --- | --- | --- | --- | --- | --- | --- | --- | --- | --- | --- | --- | --- | --- | --- |
| Aripiprazole | NA | -0.156(-0.471,0.144) | 0.334(-0.003,0.698) | -0.801(-1.249,-0.339) | -0.133(-0.424,0.165) | -0.381(-0.725,-0.023) | -0.048(-0.337,0.23) | 0.138(-0.285,0.535) | 0.184(-0.087,0.459) | -0.01(-0.241,0.22) | -0.436(-0.608,-0.257) | -0.099(-0.367,0.157) | 0.162(-0.109,0.421) | -0.369(-0.778,0.019) | -0.272(-0.582,0.016) |
| Asenapine | 0.156(-0.144,0.471) | NA | 0.49(0.099,0.886) | -0.645(-1.125,-0.159) | 0.023(-0.307,0.371) | -0.225(-0.596,0.179) | 0.109(-0.225,0.431) | 0.294(-0.141,0.728) | 0.34(0.046,0.653) | 0.146(-0.158,0.476) | -0.28(-0.513,-0.029) | 0.057(-0.275,0.392) | 0.318(-0.001,0.637) | -0.213(-0.656,0.209) | -0.116(-0.455,0.229) |
| Clozapine | -0.334(-0.698,0.003) | -0.49(-0.886,-0.099) | NA | -1.135(-1.645,-0.619) | -0.466(-0.833,-0.102) | -0.714(-1.139,-0.295) | -0.381(-0.757,-0.001) | -0.196(-0.664,0.273) | -0.15(-0.497,0.192) | -0.344(-0.716,0.031) | -0.769(-1.095,-0.465) | -0.433(-0.806,-0.073) | -0.171(-0.551,0.188) | -0.703(-1.186,-0.245) | -0.606(-0.997,-0.227) |
| Fluphenazine | 0.801(0.339,1.249) | 0.645(0.159,1.125) | 1.135(0.619,1.645) | NA | 0.668(0.217,1.107) | 0.42(-0.083,0.934) | 0.754(0.27,1.241) | 0.939(0.346,1.506) | 0.985(0.536,1.456) | 0.791(0.327,1.282) | 0.365(-0.049,0.797) | 0.702(0.23,1.189) | 0.963(0.489,1.441) | 0.432(-0.104,0.957) | 0.529(0.055,1.011) |
| Haloperidol | 0.133(-0.165,0.424) | -0.023(-0.371,0.307) | 0.466(0.102,0.833) | -0.668(-1.107,-0.217) | NA | -0.248(-0.586,0.1) | 0.085(-0.261,0.413) | 0.271(-0.178,0.722) | 0.316(0.021,0.628) | 0.122(-0.205,0.441) | -0.303(-0.532,-0.068) | 0.034(-0.336,0.335) | 0.295(-0.036,0.594) | -0.236(-0.654,0.181) | -0.14(-0.49,0.193) |
| Loxapine | 0.381(0.023,0.725) | 0.225(-0.179,0.596) | 0.714(0.295,1.139) | -0.42(-0.934,0.083) | 0.248(-0.1,0.586) | NA | 0.333(-0.046,0.703) | 0.519(0.021,1.017) | 0.565(0.211,0.932) | 0.37(-0.02,0.749) | -0.055(-0.367,0.246) | 0.282(-0.109,0.64) | 0.543(0.177,0.889) | 0.012(-0.322,0.344) | 0.108(-0.298,0.484) |
| Lurasidone | 0.048(-0.23,0.337) | -0.109(-0.431,0.225) | 0.381(0.001,0.757) | -0.754(-1.241,-0.27) | -0.085(-0.413,0.261) | -0.333(-0.703,0.046) | NA | 0.185(-0.246,0.606) | 0.231(-0.071,0.528) | 0.037(-0.281,0.346) | -0.388(-0.612,-0.157) | -0.052(-0.374,0.257) | 0.21(-0.079,0.516) | -0.322(-0.739,0.103) | -0.225(-0.551,0.091) |
| Molindone | -0.138(-0.535,0.285) | -0.294(-0.728,0.141) | 0.196(-0.273,0.664) | -0.939(-1.506,-0.346) | -0.271(-0.722,0.178) | -0.519(-1.017,-0.021) | -0.185(-0.606,0.246) | NA | 0.046(-0.309,0.406) | -0.148(-0.555,0.275) | -0.574(-0.931,-0.186) | -0.237(-0.656,0.188) | 0.024(-0.339,0.407) | -0.507(-1.028,-0.004) | -0.41(-0.824,0.033) |
| Olanzapine | -0.184(-0.459,0.087) | -0.34(-0.653,-0.046) | 0.15(-0.192,0.497) | -0.985(-1.456,-0.536) | -0.316(-0.628,-0.021) | -0.565(-0.932,-0.211) | -0.231(-0.528,0.071) | -0.046(-0.406,0.309) | NA | -0.194(-0.483,0.103) | -0.619(-0.822,-0.414) | -0.283(-0.581,-0.003) | -0.022(-0.284,0.227) | -0.553(-0.984,-0.147) | -0.456(-0.751,-0.16) |
| Paliperidone | 0.01(-0.22,0.241) | -0.146(-0.476,0.158) | 0.344(-0.031,0.716) | -0.791(-1.282,-0.327) | -0.122(-0.441,0.205) | -0.37(-0.749,0.02) | -0.037(-0.346,0.281) | 0.148(-0.275,0.555) | 0.194(-0.103,0.483) | NA | -0.425(-0.633,-0.211) | -0.089(-0.383,0.201) | 0.173(-0.119,0.466) | -0.359(-0.773,0.041) | -0.262(-0.574,0.05) |
| Placebo | 0.436(0.257,0.608) | 0.28(0.029,0.513) | 0.769(0.465,1.095) | -0.365(-0.797,0.049) | 0.303(0.068,0.532) | 0.055(-0.246,0.367) | 0.388(0.157,0.612) | 0.574(0.186,0.931) | 0.619(0.414,0.822) | 0.425(0.211,0.633) | NA | 0.337(0.13,0.546) | 0.598(0.386,0.8) | 0.067(-0.303,0.432) | 0.163(-0.067,0.385) |
| Quetiapine | 0.099(-0.157,0.367) | -0.057(-0.392,0.275) | 0.433(0.073,0.806) | -0.702(-1.189,-0.23) | -0.034(-0.335,0.336) | -0.282(-0.64,0.109) | 0.052(-0.257,0.374) | 0.237(-0.188,0.656) | 0.283(0.003,0.581) | 0.089(-0.201,0.383) | -0.337(-0.546,-0.13) | NA | 0.261(-0.019,0.529) | -0.27(-0.69,0.198) | -0.173(-0.471,0.128) |
| Risperidone | -0.162(-0.421,0.109) | -0.318(-0.637,0.001) | 0.171(-0.188,0.551) | -0.963(-1.441,-0.489) | -0.295(-0.594,0.036) | -0.543(-0.889,-0.177) | -0.21(-0.516,0.079) | -0.024(-0.407,0.339) | 0.022(-0.227,0.284) | -0.173(-0.466,0.119) | -0.598(-0.8,-0.386) | -0.261(-0.529,0.019) | NA | -0.531(-0.928,-0.115) | -0.434(-0.726,-0.123) |
| Trifluoperazine | 0.369(-0.019,0.778) | 0.213(-0.209,0.656) | 0.703(0.245,1.186) | -0.432(-0.957,0.104) | 0.236(-0.181,0.654) | -0.012(-0.344,0.322) | 0.322(-0.103,0.739) | 0.507(0.004,1.028) | 0.553(0.147,0.984) | 0.359(-0.041,0.773) | -0.067(-0.432,0.303) | 0.27(-0.198,0.69) | 0.531(0.115,0.928) | NA | 0.097(-0.344,0.524) |
| Ziprasidone | 0.272(-0.016,0.582) | 0.116(-0.229,0.455) | 0.606(0.227,0.997) | -0.529(-1.011,-0.055) | 0.14(-0.193,0.49) | -0.108(-0.484,0.298) | 0.225(-0.091,0.551) | 0.41(-0.033,0.824) | 0.456(0.16,0.751) | 0.262(-0.05,0.574) | -0.163(-0.385,0.067) | 0.173(-0.128,0.471) | 0.434(0.123,0.726) | -0.097(-0.524,0.344) | NA |

**Appendix Table 15:** League table for all comparisons in CA network. Informative priors from GP studies using expert’s opinion for β and moderate downweight for GP studies with high RoB

|  | Aripiprazole | Asenapine | Clozapine | Fluphenazine | Haloperidol | Loxapine | Lurasidone | Molindone | Olanzapine | Paliperidone | Placebo | Quetiapine | Risperidone | Trifluoperazine | Ziprasidone |
| --- | --- | --- | --- | --- | --- | --- | --- | --- | --- | --- | --- | --- | --- | --- | --- |
| Aripiprazole | NA | -0.159(-0.456,0.129) | 0.322(-0.019,0.657) | -0.824(-1.289,-0.374) | -0.132(-0.433,0.184) | -0.323(-0.658,0.023) | -0.06(-0.337,0.21) | 0.105(-0.275,0.472) | 0.18(-0.085,0.446) | 0(-0.214,0.232) | -0.435(-0.604,-0.259) | -0.103(-0.33,0.133) | 0.158(-0.111,0.431) | -0.333(-0.752,0.054) | -0.26(-0.529,0.004) |
| Asenapine | 0.159(-0.129,0.456) | NA | 0.481(0.097,0.873) | -0.665(-1.133,-0.21) | 0.027(-0.297,0.353) | -0.163(-0.541,0.202) | 0.099(-0.237,0.43) | 0.265(-0.136,0.662) | 0.34(0.034,0.641) | 0.16(-0.165,0.496) | -0.276(-0.502,-0.029) | 0.057(-0.242,0.368) | 0.317(0.017,0.635) | -0.174(-0.61,0.243) | -0.101(-0.4,0.214) |
| Clozapine | -0.322(-0.657,0.019) | -0.481(-0.873,-0.097) | NA | -1.146(-1.64,-0.681) | -0.454(-0.8,-0.091) | -0.645(-1.05,-0.225) | -0.382(-0.746,-0.026) | -0.217(-0.635,0.197) | -0.142(-0.469,0.191) | -0.322(-0.665,0.039) | -0.757(-1.043,-0.456) | -0.425(-0.762,-0.068) | -0.164(-0.508,0.201) | -0.655(-1.126,-0.217) | -0.582(-0.933,-0.216) |
| Fluphenazine | 0.824(0.374,1.289) | 0.665(0.21,1.133) | 1.146(0.681,1.64) | NA | 0.692(0.251,1.131) | 0.502(0.017,1.019) | 0.764(0.311,1.226) | 0.93(0.402,1.452) | 1.005(0.559,1.464) | 0.825(0.383,1.288) | 0.389(-0.022,0.8) | 0.722(0.287,1.181) | 0.982(0.535,1.447) | 0.491(-0.018,1.033) | 0.564(0.119,1.011) |
| Haloperidol | 0.132(-0.184,0.433) | -0.027(-0.353,0.297) | 0.454(0.091,0.8) | -0.692(-1.131,-0.251) | NA | -0.191(-0.535,0.141) | 0.072(-0.261,0.386) | 0.238(-0.148,0.634) | 0.312(0.024,0.6) | 0.132(-0.16,0.451) | -0.303(-0.544,-0.065) | 0.029(-0.287,0.336) | 0.29(-0.005,0.586) | -0.201(-0.608,0.174) | -0.128(-0.455,0.187) |
| Loxapine | 0.323(-0.023,0.658) | 0.163(-0.202,0.541) | 0.645(0.225,1.05) | -0.502(-1.019,-0.017) | 0.191(-0.141,0.535) | NA | 0.263(-0.101,0.615) | 0.428(-0.027,0.864) | 0.503(0.153,0.844) | 0.323(-0.031,0.677) | -0.113(-0.414,0.173) | 0.22(-0.146,0.569) | 0.48(0.113,0.827) | -0.011(-0.327,0.312) | 0.063(-0.31,0.431) |
| Lurasidone | 0.06(-0.21,0.337) | -0.099(-0.43,0.237) | 0.382(0.026,0.746) | -0.764(-1.226,-0.311) | -0.072(-0.386,0.261) | -0.263(-0.615,0.101) | NA | 0.166(-0.219,0.558) | 0.241(-0.049,0.533) | 0.061(-0.215,0.365) | -0.375(-0.574,-0.159) | -0.043(-0.315,0.262) | 0.218(-0.066,0.521) | -0.273(-0.678,0.122) | -0.2(-0.503,0.11) |
| Molindone | -0.105(-0.472,0.275) | -0.265(-0.662,0.136) | 0.217(-0.197,0.635) | -0.93(-1.452,-0.402) | -0.238(-0.634,0.148) | -0.428(-0.864,0.027) | -0.166(-0.558,0.219) | NA | 0.075(-0.262,0.394) | -0.105(-0.491,0.302) | -0.541(-0.873,-0.196) | -0.208(-0.588,0.179) | 0.052(-0.294,0.4) | -0.439(-0.922,0.041) | -0.365(-0.774,0.055) |
| Olanzapine | -0.18(-0.446,0.085) | -0.34(-0.641,-0.034) | 0.142(-0.191,0.469) | -1.005(-1.464,-0.559) | -0.312(-0.6,-0.024) | -0.503(-0.844,-0.153) | -0.241(-0.533,0.049) | -0.075(-0.394,0.262) | NA | -0.18(-0.454,0.104) | -0.616(-0.804,-0.414) | -0.283(-0.536,-0.008) | -0.023(-0.267,0.224) | -0.514(-0.934,-0.108) | -0.44(-0.732,-0.161) |
| Paliperidone | 0(-0.232,0.214) | -0.16(-0.496,0.165) | 0.322(-0.039,0.665) | -0.825(-1.288,-0.383) | -0.132(-0.451,0.16) | -0.323(-0.677,0.031) | -0.061(-0.365,0.215) | 0.105(-0.302,0.491) | 0.18(-0.104,0.454) | NA | -0.436(-0.648,-0.229) | -0.103(-0.379,0.174) | 0.157(-0.118,0.436) | -0.334(-0.756,0.049) | -0.26(-0.554,0.026) |
| Placebo | 0.435(0.259,0.604) | 0.276(0.029,0.502) | 0.757(0.456,1.043) | -0.389(-0.8,0.022) | 0.303(0.065,0.544) | 0.113(-0.173,0.414) | 0.375(0.159,0.574) | 0.541(0.196,0.873) | 0.616(0.414,0.804) | 0.436(0.229,0.648) | NA | 0.333(0.145,0.531) | 0.593(0.401,0.786) | 0.102(-0.269,0.448) | 0.175(-0.038,0.401) |
| Quetiapine | 0.103(-0.133,0.33) | -0.057(-0.368,0.242) | 0.425(0.068,0.762) | -0.722(-1.181,-0.287) | -0.029(-0.336,0.287) | -0.22(-0.569,0.146) | 0.043(-0.262,0.315) | 0.208(-0.179,0.588) | 0.283(0.008,0.536) | 0.103(-0.174,0.379) | -0.333(-0.531,-0.145) | NA | 0.26(-0.017,0.516) | -0.231(-0.639,0.155) | -0.157(-0.443,0.134) |
| Risperidone | -0.158(-0.431,0.111) | -0.317(-0.635,-0.017) | 0.164(-0.201,0.508) | -0.982(-1.447,-0.535) | -0.29(-0.586,0.005) | -0.48(-0.827,-0.113) | -0.218(-0.521,0.066) | -0.052(-0.4,0.294) | 0.023(-0.224,0.267) | -0.157(-0.436,0.118) | -0.593(-0.786,-0.401) | -0.26(-0.516,0.017) | NA | -0.491(-0.886,-0.099) | -0.418(-0.702,-0.116) |
| Trifluoperazine | 0.333(-0.054,0.752) | 0.174(-0.243,0.61) | 0.655(0.217,1.126) | -0.491(-1.033,0.018) | 0.201(-0.174,0.608) | 0.011(-0.312,0.327) | 0.273(-0.122,0.678) | 0.439(-0.041,0.922) | 0.514(0.108,0.934) | 0.334(-0.049,0.756) | -0.102(-0.448,0.269) | 0.231(-0.155,0.639) | 0.491(0.099,0.886) | NA | 0.073(-0.315,0.494) |
| Ziprasidone | 0.26(-0.004,0.529) | 0.101(-0.214,0.4) | 0.582(0.216,0.933) | -0.564(-1.011,-0.119) | 0.128(-0.187,0.455) | -0.063(-0.431,0.31) | 0.2(-0.11,0.503) | 0.365(-0.055,0.774) | 0.44(0.161,0.732) | 0.26(-0.026,0.554) | -0.175(-0.401,0.038) | 0.157(-0.134,0.443) | 0.418(0.116,0.702) | -0.073(-0.494,0.315) | NA |

**Appendix Table 16:** League table for all comparisons in CA network. Informative priors from GP studies using expert’s opinion for $\beta$ and moderate downweight for GP studies evaluating interventions in $T_{\alpha}-T_{c}$.

**Appendix Table 17:** League table for all comparisons in CA network using a NMA model with non-informative priors.

|  | Aripiprazole | Asenapine | Clozapine | Fluphenazine | Haloperidol | Loxapine | Lurasidone | Molindone | Olanzapine | Paliperidone | Placebo | Quetiapine | Risperidone | Trifluoperazine | Ziprasidone |
| --- | --- | --- | --- | --- | --- | --- | --- | --- | --- | --- | --- | --- | --- | --- | --- |
| Aripiprazole | NA | -0.046(-0.517,0.44) | 0.628(-0.142,1.404) | -1.555(-2.581,-0.524) | -0.193(-0.927,0.525) | -0.295(-1.284,0.633) | 0.05(-0.388,0.512) | 0.361(-0.212,0.97) | 0.317(-0.149,0.769) | -0.036(-0.342,0.282) | -0.433(-0.718,-0.137) | -0.044(-0.381,0.293) | 0.24(-0.2,0.66) | -0.259(-1.383,0.801) | -0.281(-0.744,0.224) |
| Asenapine | 0.046(-0.44,0.517) | NA | 0.674(-0.084,1.466) | -1.509(-2.529,-0.474) | -0.147(-0.916,0.621) | -0.249(-1.254,0.7) | 0.096(-0.434,0.608) | 0.407(-0.234,1.044) | 0.362(-0.16,0.863) | 0.01(-0.477,0.519) | -0.387(-0.762,-0.011) | 0.001(-0.471,0.515) | 0.286(-0.262,0.763) | -0.213(-1.321,0.908) | -0.235(-0.756,0.294) |
| Clozapine | -0.628(-1.404,0.142) | -0.674(-1.466,0.084) | NA | -2.183(-3.109,-1.171) | -0.821(-1.519,-0.16) | -0.923(-1.823,-0.014) | -0.578(-1.352,0.227) | -0.267(-1.009,0.509) | -0.312(-0.917,0.308) | -0.664(-1.439,0.154) | -1.061(-1.731,-0.351) | -0.673(-1.415,0.09) | -0.388(-1.072,0.31) | -0.887(-1.869,0.091) | -0.909(-1.721,-0.094) |
| Fluphenazine | 1.555(0.524,2.581) | 1.509(0.474,2.529) | 2.183(1.171,3.109) | NA | 1.362(0.642,2.089) | 1.26(0.329,2.21) | 1.604(0.555,2.706) | 1.916(0.877,2.953) | 1.871(0.958,2.833) | 1.518(0.502,2.58) | 1.122(0.12,2.152) | 1.51(0.51,2.561) | 1.795(0.828,2.733) | 1.296(0.265,2.343) | 1.274(0.223,2.373) |
| Haloperidol | 0.193(-0.525,0.927) | 0.147(-0.621,0.916) | 0.821(0.16,1.519) | -1.362(-2.089,-0.642) | NA | -0.102(-0.718,0.535) | 0.243(-0.507,1.026) | 0.554(-0.186,1.301) | 0.509(-0.046,1.115) | 0.157(-0.605,0.938) | -0.24(-0.892,0.442) | 0.148(-0.577,0.902) | 0.433(-0.191,1.078) | -0.066(-0.812,0.705) | -0.088(-0.863,0.684) |
| Loxapine | 0.295(-0.633,1.284) | 0.249(-0.7,1.254) | 0.923(0.014,1.823) | -1.26(-2.21,-0.329) | 0.102(-0.535,0.718) | NA | 0.345(-0.618,1.372) | 0.656(-0.285,1.616) | 0.611(-0.203,1.455) | 0.259(-0.702,1.243) | -0.138(-1.033,0.834) | 0.251(-0.684,1.232) | 0.535(-0.337,1.448) | 0.036(-0.413,0.494) | 0.014(-0.953,1.018) |
| Lurasidone | -0.05(-0.512,0.388) | -0.096(-0.608,0.434) | 0.578(-0.227,1.352) | -1.604(-2.706,-0.555) | -0.243(-1.026,0.507) | -0.345(-1.372,0.618) | NA | 0.311(-0.324,0.948) | 0.267(-0.28,0.78) | -0.086(-0.579,0.426) | -0.482(-0.85,-0.112) | -0.094(-0.58,0.393) | 0.19(-0.358,0.677) | -0.309(-1.437,0.779) | -0.331(-0.852,0.186) |
| Molindone | -0.361(-0.97,0.212) | -0.407(-1.044,0.234) | 0.267(-0.509,1.009) | -1.916(-2.953,-0.877) | -0.554(-1.301,0.186) | -0.656(-1.616,0.285) | -0.311(-0.948,0.324) | NA | -0.045(-0.509,0.41) | -0.397(-0.992,0.199) | -0.794(-1.316,-0.258) | -0.406(-0.985,0.196) | -0.121(-0.602,0.327) | -0.62(-1.655,0.444) | -0.642(-1.265,0.037) |
| Olanzapine | -0.317(-0.769,0.149) | -0.362(-0.863,0.16) | 0.312(-0.308,0.917) | -1.871(-2.833,-0.958) | -0.509(-1.115,0.046) | -0.611(-1.455,0.203) | -0.267(-0.78,0.28) | 0.045(-0.41,0.509) | NA | -0.353(-0.856,0.176) | -0.749(-1.119,-0.342) | -0.361(-0.819,0.119) | -0.076(-0.431,0.285) | -0.575(-1.516,0.392) | -0.597(-1.127,-0.04) |
| Paliperidone | 0.036(-0.282,0.342) | -0.01(-0.519,0.477) | 0.664(-0.154,1.439) | -1.518(-2.58,-0.502) | -0.157(-0.938,0.605) | -0.259(-1.243,0.702) | 0.086(-0.426,0.579) | 0.397(-0.199,0.992) | 0.353(-0.176,0.856) | NA | -0.396(-0.757,-0.066) | -0.008(-0.461,0.406) | 0.276(-0.213,0.738) | -0.223(-1.302,0.872) | -0.245(-0.764,0.289) |
| Placebo | 0.433(0.137,0.718) | 0.387(0.011,0.762) | 1.061(0.351,1.731) | -1.122(-2.152,-0.12) | 0.24(-0.442,0.892) | 0.138(-0.834,1.033) | 0.482(0.112,0.85) | 0.794(0.258,1.316) | 0.749(0.342,1.119) | 0.396(0.066,0.757) | NA | 0.388(0.08,0.705) | 0.673(0.305,1.001) | 0.174(-0.868,1.224) | 0.152(-0.226,0.536) |
| Quetiapine | 0.044(-0.293,0.381) | -0.001(-0.515,0.471) | 0.673(-0.09,1.415) | -1.51(-2.561,-0.51) | -0.148(-0.902,0.577) | -0.251(-1.232,0.684) | 0.094(-0.393,0.58) | 0.406(-0.196,0.985) | 0.361(-0.119,0.819) | 0.008(-0.406,0.461) | -0.388(-0.705,-0.08) | NA | 0.285(-0.162,0.696) | -0.214(-1.297,0.857) | -0.236(-0.718,0.288) |
| Risperidone | -0.24(-0.66,0.2) | -0.286(-0.763,0.262) | 0.388(-0.31,1.072) | -1.795(-2.733,-0.828) | -0.433(-1.078,0.191) | -0.535(-1.448,0.337) | -0.19(-0.677,0.358) | 0.121(-0.327,0.602) | 0.076(-0.285,0.431) | -0.276(-0.738,0.213) | -0.673(-1.001,-0.305) | -0.285(-0.696,0.162) | NA | -0.499(-1.479,0.518) | -0.521(-1.011,0.009) |
| Trifluoperazine | 0.259(-0.801,1.383) | 0.213(-0.908,1.321) | 0.887(-0.091,1.869) | -1.296(-2.343,-0.265) | 0.066(-0.705,0.812) | -0.036(-0.494,0.413) | 0.309(-0.779,1.437) | 0.62(-0.444,1.655) | 0.575(-0.392,1.516) | 0.223(-0.872,1.302) | -0.174(-1.224,0.868) | 0.214(-0.857,1.297) | 0.499(-0.518,1.479) | NA | -0.022(-1.114,1.079) |
| Ziprasidone | 0.281(-0.224,0.744) | 0.235(-0.294,0.756) | 0.909(0.094,1.721) | -1.274(-2.373,-0.223) | 0.088(-0.684,0.863) | -0.014(-1.018,0.953) | 0.331(-0.186,0.852) | 0.642(-0.037,1.265) | 0.597(0.04,1.127) | 0.245(-0.289,0.764) | -0.152(-0.536,0.226) | 0.236(-0.288,0.718) | 0.521(-0.009,1.011) | 0.022(-1.079,1.114) | NA |

**References**

1. Law M, Alam N, Veroniki AA, Yu Y, Jackson D. Two new approaches for the visualisation of models for network meta-analysis. *BMC Med Res Methodol*. 2019;19(1):61. doi:10.1186/s12874-019-0689-9
