# Supplementary material for: Sharing information across patient subgroups to draw conclusions from sparse treatment networks
Source: Biom J. Author manuscript; Available in PMC 2024 Dec 20. (PMC7617248; doi:10.1002/bimj.202200316)
Supplement: Supplementary Material [file EMS201286-supplement-Supplementary_Material.zip › bimj2569-sup-0003-suppmat.zip!/README.pdf]

TITLE: "Sharing information across patient subgroups to draw conclusions from sparse treatment networks"

AUTHORS: Theodoros Evrenoglou, Silvia Metelli, Johannes-Schneider Thomas, Spyridon Siafis, Rebecca Turner, Stefan Leucht, Anna Chaimani

MAIN AUTHOR OF THE CODE: Theodoros Evrenoglou, tevrenoglou@gmail.com

R version 4.0.3 (2020-10-10)

Platform: x86_64-w64-mingw32/x64 (64-bit)

Running under: Windows 10 x64 (build 19045)

Matrix products: default

locale:

[1] LC_COLLATE=English_United States.1252 LC_CTYPE=English_United States.1252 LC_MONETARY=English_United States.1252

[4] LC_NUMERIC=C LC_TIME=English_United States.1252

attached base packages:

[1] stats graphics grDevices utils datasets methods base

other attached packages:

[1] gplots_3.1.3 igraph_1.2.6 mvtnorm_1.1-1 ggsci_2.9 remotes_2.2.0 nmajags_0.1-0

[7] devtools_2.3.2 usethis_1.6.3 curl_5.0.0 R2jags_0.6-1 rjags_4-10 coda_0.19-4

[13] netmeta_2.1-0 meta_6.2-1 forcats_0.5.0 stringr_1.4.0 dplyr_1.0.10 purrr_0.3.4

[19] readr_1.4.0 tidyr_1.1.2 tibble_3.1.8 ggplot2_3.3.5 tidyverse_1.3.0 readxl_1.3.1

loaded via a namespace (and not attached):

[1] nlme_3.1-149 bitops_1.0-7 fs_1.5.2 lubridate_1.7.9.2 httr_1.4.2 rprojroot_2.0.2

[7] tools_4.0.3 backports_1.2.0 utf8_1.1.4 R6_2.5.0 R2WinBUGS_2.1-21 metafor_3.0-2

[13] KernSmooth_2.23-17 DBI_1.1.0 colorspace_2.0-0 withr_2.5.0 tidyselect_1.2.0

[18] prettyunits_1.1.1 processx_3.4.4 compiler_4.0.3 cli_3.6.0 rvest_0.3.6 xml2_1.3.2

[24] desc_1.2.0 caTools_1.18.0 scales_1.2.1 callr_3.5.1 digest_0.6.27

[29] minqa_1.2.4 pkgconfig_2.0.3 lme4_1.1-27.1 sessioninfo_1.1.1 dbplyr_2.0.0

[34] rlang_1.0.6 rstudioapi_0.13 generics_0.1.0 jsonlite_1.7.1 gtools_3.8.2 magrittr_2.0.3

[40] Matrix_1.2-18 Rcpp_1.0.10 munsell_0.5.0 fansi_0.4.1 abind_1.4-5 lifecycle_1.0.3

[46] stringi_1.5.3 CompQuadForm_1.4.3 mathjaxr_1.0-1 MASS_7.3-53 pkgbuild_1.1.0

[51] grid_4.0.3 parallel_4.0.3 crayon_1.3.4 lattice_0.20-41 haven_2.3.1 splines_4.0.3
[57] hms_0.5.3 ps_1.4.0 pillar_1.8.1 boot_1.3-25 pkgload_1.1.0 magic_1.5-9
[63] reprex_0.3.0 glue_1.6.2 modelr_0.1.8 vctrs_0.5.1 nloptr_1.2.2.2 testthat_3.0.4
[69] cellranger_1.1.0 gtable_0.3.0 assertthat_0.2.1 broom_0.7.9 memoise_1.1.0
[74] ellipsis_0.3.2

INSTRUCTIONS:

Access: All the files described in this document can also be accessed through the public GitHub repository at: https://github.com/TEvrenoglou/codes_sharing_information. Through this link you can download the *Data_and_Codes* folder in your personal device.

Code execution process:

1. Set the folder *Data_and_Codes* as the current working directory
2. Run the **master**.**R** script in the **Codes** folder. This will reproduce all the figures and tables in the main manuscript and in the Appendix that were constructed using R code. **Figure 1** of the main manuscript was created using the online tool: https://www.nmastudioapp.com/ and thus there is no code to reproduce this figure. **Figure 1** though does not involve any numerical results but only a graphical representation of the data. In addition, the numerical values that each line represents in **Figure 2** of the main manuscript were added externally without using any code. Please, note that the `nmajags` library used in our code, is currently available only through GitHub. Please make sure to install it using the code:

```
library(remotes)
remotes::install_github("guido-s/nmajags")
```

Folder **Codes**: It contains both **R** and **JAGS** code

1. **helpers.R**, contains R functions to facilitate the analysis performed through the **master.R** file.
2. **master.R**, uses the functions defined in the **helpers.R**, performs all the analyses, reproduces tables and figures and automatically saves them in the **Results** folder.

The following files are included in the **Code** folder and contain **JAGS** codes.

- **NMA_model_data_based_beta.R**, contains the JAGS code used for the first stage of the proposed approach. It performs network meta-analysis with data based priors for beta.

- **NMA_model_expert_based_beta.R**, contains the JAGS code used for the first stage of the proposed approach. It performs network meta-analysis with expert opinion based priors for beta.
- **NMA_model_node_splitting.R**, contains the JAGS code to fit the NMA node-splitting model.
- **NMA_model_with_informative_priors.R**, contains the JAGS code used for the second stage of the proposed approach. It performs network meta-analysis with the informative priors constructed at the first stage.
- **pairwiseMA_data_expert_based_priors.R**, contains the JAGS code to fit the pairwise meta-analysis model and construct priors for the beta parameter according to the (i) data based and (ii) expert opinion based approach.
- **standard_NMA_model.R**, contains the JAGS code to perform network meta-analysis using non-informative priors.

Folder **Data** contains:

- file **data.xlsx**, contains the main dataset of the analysis.
- file **direct_evidence.xlsx**, contains pooled data related to the direct comparisons in the children and adolescent network.
- file **expert_opinion.xlsx**, contains the expert opinions used to fit the expert opinion based models.
- file **GP_pooled_data.xlsx**, contains the pooled General patient results that are used to construct expert opinion priors and to fit the models based on expert opinion.
- file **posterior_samples.xlsx,** contains density data to reproduce figures 2-6 of Appendix 1.
- file **priors.xlsx,** contains the priors to be used for the consistency checks.

Folder **Results**: It contains all the tables and figures presented in the **main manuscript** and in the **Appendix 1**, that were constructed using R code. These results are given either in "csv" or "pdf" format for tables and figures respectively. All the tables and figures included in that file can be reproduced from the **master.R** file in the **Codes** folder.

DATA AVAILABILITY: Data for the application are available through the **Data** folder.

All the paths in the files are relative, based on the assumption that the working directory is set properly as stated in the "Code execution" section.
